# A SILAC-based Approach Identifies Substrates of Caspase-dependent Cleavage upon TRAIL-induced Apoptosis*[Fn FN2]

**DOI:** 10.1074/mcp.M112.024679

**Published:** 2013-01-13

**Authors:** Gabriele Stoehr, Christoph Schaab, Johannes Graumann, Matthias Mann

**Affiliations:** From the ‡Department of Proteomics and Signal Transduction, Max-Planck Institute of Biochemistry, Am Klopferspitz 18, D-82152 Martinsried, Germany;; §Evotec (Munich) GmbH, Am Klopferspitz 19a, D-82152 Martinsried, Germany;; ¶Weill Cornell Medical College in Qatar, Qatar Foundation, Education City, Doha, State of Qatar

## Abstract

The extracellular ligand-induced extrinsic pathway of apoptosis is executed via caspase protease cascades that activate downstream effectors by means of site-directed proteolysis. Here we identify proteome changes upon the induction of apoptosis by the cytokine tumor necrosis factor–related apoptosis-inducing ligand (TRAIL) in a Jurkat T cell line. We detected caspase-dependent cleavage substrates by quantifying protein intensities before and after TRAIL induction in SDS gel slices. Apoptotic protein cleavage events are identified by a characteristic stable isotope labeling with amino acids in cell culture (SILAC) ratio pattern across gel slices that results from differential migration of the cleaved and uncleaved proteins. We applied a statistical test to define apoptotic substrates in the proteome. Our approach identified more than 650 of these cleaved proteins in response to TRAIL-induced apoptosis, including many previously unknown substrates and cleavage sites. Inhibitor treatment combined with triple SILAC demonstrated that the detected cleavage events were caspase dependent. Proteins located in the lumina of organelles such as mitochondria and endoplasmic reticulum were significantly underrepresented in the substrate population. Interestingly, caspase cleavage is generally observed in not only one but several members of stable complexes, but often with lower stoichiometry. For instance, all five proteins of the condensin I complex were cleaved upon TRAIL treatment. The apoptotic substrate proteome data can be accessed and visualized in the MaxQB database and might prove useful for basic and clinical research into TRAIL-induced apoptosis. The technology described here is extensible to a wide range of other proteolytic cleavage events.

Apoptosis is an essential cellular mechanism regulating normal physiological processes, for instance, in development (1, 2). As a specific and programmed form of cell death, apoptosis leads to a controlled disposal of cells, relying on the proteolytic cleavage of specific protein substrates as the central mechanism. Moreover, the selective induction of apoptosis plays an important role in diseases such as cancer. It is thus important to determine the proteins and pathways involved in apoptosis and to characterize their relevance for induction of the pathway by different stimuli. The main players in apoptosis are caspases (Cys-dependent Asp-specific proteases), which specifically cleave C-terminally of an aspartate and are represented by 11 functional genes in the human genome. Auto-cleavage of upstream caspases in response to the induction of apoptosis initiates a cascade of downstream cleavage events resulting in activation or inactivation, as well as translocation, of these substrates (3–6).

In recent years, several methods have been introduced to identify substrates cleaved in a caspase-dependent manner, as well as the exact location of cleavage sites. *In vitro* approaches such as the incubation of peptides or protein libraries with the active protease of interest have led to the identification of substrate motifs but do not necessarily represent *in vivo* events in the context of an intact cell (7). Mass spectrometry (MS)-based methods employed may be divided into those directed toward the detection of the peptides cleaved by the protease and those applied at the proteome level for the identification of substrates and downstream effects. The former methods generally chemically block all preexisting N-termini and subsequently take advantage of the *de novo* generation of N-termini by the protease, which serves as a handle to enrich and detect the corresponding peptide. The original method in this class was N-terminal COFRADIC (8, 9), and it was used to detect Fas-induced cleavage events in Jurkat T cells (10). Recent peptide-selective methods include the exclusive labeling of novel N-termini by biotin followed by peptide capture on avidin columns (11), but others, using both positive and negative selection strategies, have also been described (12–15). Peptide-based methods have the advantage of allowing direct identification of the cleavage site. Information about the substrate protein, however, is limited to a single peptide per cleavage event, and this peptide might not be readily detectable.

Global approaches applied at the proteome level, in contrast, attempt to identify as many cleaved proteins as possible without necessarily determining the exact cleavage site within the protein (16, 17). In many cases, two-dimensional gel electrophoresis has been used to differentiate cleaved and uncleaved protein states (18–20). This approach, however, lacks reproducibility, sensitivity, and throughput. A pioneering study based on one-dimensional SDS page identified several previously known cleaved proteins in Fas-induced Jurkat T cells by their molecular mass shift, yet it remained small in scale (21). The approach was further extended to include the quantitative ratio information of the proteins in combination with offline LC-MALDI-MS/MS (22).

Building on these studies, Cravatt and coworkers extended a global approach that takes into account information about molecular weight differences between uncleaved and corresponding cleaved proteins, termed PROTOMAP (23). Identified peptides were mapped onto the protein sequences to provide information about the location of the cleavage products. Because label-free quantitation is used and samples consequently are processed in parallel, the approach is labor intensive and requires high reproducibility of the experiments.

In an attempt to circumvent the limitations of the methods mentioned above, we set out to develop a quantitative method based on stable isotope labeling with amino acids in cell culture (SILAC)^1^ for the identification of apoptosis-dependent substrates of protein cleavage on the proteome level, but making use of accurate peptide-based ratio information for each substrate protein. In this way, we aimed to combine advantages of each of the above approaches. Furthermore, we included in our analysis a combination of a high-resolution LC-MS/MS workflow with stringent statistical analysis of the results.

Several previous proteomics studies of apoptosis have investigated the effects of *intrinsic* stimuli. Despite its importance for the development of cancer therapeutics, no global proteome study has characterized cleavage events induced by the *extrinsic* stimulus tumor necrosis factor–related apoptosis-inducing ligand (TRAIL), apart from one targeted study focusing on cleavage kinetics (24). In that study, TRAIL-treated (extrinsic) and staurosporine-treated (intrinsic) samples were compared via the selected reaction monitoring of ∼1,000 caspase-derived peptides. In our study, we performed triple SILAC labeling in the well-established system of TRAIL-induced apoptosis in Jurkat T cells, which allowed us to validate the caspase dependence of the detected cleavage events. Our study sheds light on the fate of protein complexes in cell death and provides the first in-depth overview of cleaved substrates in response to extrinsic TRAIL-induced apoptosis.

During the preparation of this manuscript, a report by the Cravatt group extended their PROTOMAP approach (23) using SILAC, making it quantitative (25). That study elegantly extracts information on the crosstalk between phosphorylation events in the cleavage motifs and the regulation of the proteolytic pathway in apoptosis. It thereby underscores the necessity and potential of quantitative approaches for the study of proteolytic events in a global manner.

## EXPERIMENTAL PROCEDURES

### 

#### 

##### Evaluation of TRAIL Treatment and Inhibitor Concentrations

Jurkat T cells (0.4 × 10^6^ cells/ml) were treated with 50 ng/ml, 100 ng/ml, 200 ng/ml, or 500 ng/ml human TRAIL Apo-II ligand (PeproTech, Inc., Rocky Hill, NJ; 50 μg, stock 100 ng/μl) or mock and incubated at 37 °C in 5% CO_2_ for 24 h. Cell growth was regularly examined, and cell morphology was checked using 5-h light microscopy and a cell counter (Countess, Invitrogen, Life Technologies, Grand Island, NY). Based on these experiments, a concentration of 100 ng/ml was selected for further analyses.

Jurkat T cells were treated with varying concentrations of the pan-caspase inhibitor z-VAD-FMK (0.1 μm, 1 μm, 10 μm, and 50 μm in 2 ml (0.75 × 10^6^ cells/ml); R&D Systems, Minneapolis, MN; 1 mg) 10 min prior to 5 h of incubation with 100 ng/ml of either TRAIL or mock. Cells were washed twice with ice-cold PBS, and cell pellets were frozen at −80 °C until further analysis using Western blotting.

##### Flow Cytometry Analysis

For the detection of apoptotic and necrotic cells, the Annexin V-FITC Kit (Apoptosis Detection Kit, Beckman Coulter, Krefeld, Germany) was used. Cells were washed and resuspend in ice-cold water. An antibody mix for propidium iodide/Annexin V-FITC (1:20 propidium iodide and 1:100 Annexin-V FITC) was prepared and added to the cells in Binding Buffer. Cells were kept on ice until flow cytometric analysis. Cells were analyzed after 0, 1, 2, 4, and 5 h of TRAIL induction.

##### SILAC Labeling

Jurkat T cells were cultured in RPMI medium (high-glucose GlutaMAX^TM^ devoid of arginine and lysine; Invitrogen) supplemented with 10% dialyzed fetal bovine serum (FBS) (Invitrogen) (10 kDa cut-off) and 1X penicillin/streptomycin. For SILAC labeling, arginine and lysine were added in either light (Arg0; Lys0) or heavy (Arg10; Lys8) forms to a final concentration of 33.6 μg/ml for arginine and 73 μg/ml for lysine. For triple labeling, cells were additionally cultured in medium containing Arg6 and LysD4 using the same concentrations. l-arginine (Arg0), l-lysine (Lys0), l-^13^C_6_-arginine (Arg6), l-D_4_-lysine (LysD4), l-^13^C_6_^15^N_4_-arginine (Arg10), and l-^13^C_6_^15^N_2_-lysine (Lys8) were purchased from Sigma-Aldrich. Prior to treatment, Jurkat T cells were grown for 8 to 10 passages in SILAC medium and tested for full incorporation.

##### Treatment with TRAIL

Equal numbers of Jurkat T cells (0.4 to 0.6 × 10^6^ cells/ml) were treated for 5 h with 100 ng/ml TRAIL or mock in heavy and light medium. For the inhibition study, medium labeled cells were additionally pre-treated for 10 min with 50 μm z-VAD-FMK. TRAIL treatment was stopped by the addition of ice-cold PBS to the cells. Cell suspensions were centrifuged (5 min, 4 °C, 400 *g*), and the supernatant was discarded. Cell pellets from the corresponding heavy and light cultures (or heavy, medium, and light for triple-labeling experiments) were resuspended in a small aliquot of ice-cold PBS and combined in one tube. Cells were once more washed with ice-cold PBS and centrifuged. Cell pellets were shock-frozen in liquid nitrogen and stored at −80 °C.

##### Cell Lysis and Protein Digestion

Proteins were extracted and digested following the first steps of the filter-aided sample preparation protocol (26). Cells were lysed with 4% SDS and 0.1 m DTT in 100 mm Tris/HCl pH 7.6, followed by incubation for 5 min at 95 °C. Lysates were sonicated and cleared via centrifugation at high speed (16,000 × *g*). Protein concentrations were measured using a tryptophan-fluorescence assay. 150 μg of the samples were used for further analyses. Proteins were reduced with DTT (10 mm) for 45 min at room temperature, and reduction was followed by alkylation for 30 min in the dark (55 mm iodoacetamide).

Proteins were mixed with LDS sample buffer (final: 1x; Invitrogen) and samples were boiled at 70 °C for 10 min. 50 μg of protein per sample were loaded on a polyacrylamide gel (Bis-Tris Gel, Invitrogen, 4%–12%, MOPS; 10 pockets) in each of three adjacent lanes (total of 150 μg per sample). Proteins were separated with 180 V for 45 min. Proteins were fixed and stained in the gel using standard protocols (Colloidal Blue Staining Kit, Invitrogen).

The gel was cut into between 28 and 36 slices, and corresponding slices from the three lanes were combined in one tube. Proteins in the gel were digested via standard in-gel protocols (27). Briefly, gel pieces were destained in consecutive wash steps and dehydrated using 50% and 100% ethanol. Gel pieces were dried in a SpeedVac concentrator for 5 min. Proteins were digested via the addition of sequencing-grade modified trypsin (12.9 ng/ml; Promega, Madison, WI) to the gel pieces followed by an overnight incubation at 37 °C. Peptides were subsequently extracted from the gel pieces using increasing concentrations of acetonitrile (30% to 100% acetonitrile) in separate steps. Organic solvent was removed in a SpeedVac concentrator, and peptides were desalted on reversed-phase C_18_ StageTips (Empore disk (28)) prior to LC-MS/MS analysis.

##### Western Blot Experiments

Cell pellets were lysed and proteins were separated via SDS-PAGE as mentioned before. As positive and negative controls, 10- to 15-μl Jurkat apoptosis cell lysates (etoposide-treated and untreated; Cell Signaling, Danvers, MA) were used. For CASP8 detection, 12% NuPAGE Bis-Tris Gels (Invitrogen) were used. For PARP1 analysis, 4%–12% Bis-Tris gels were used. All gels were run with MOPS buffer. Proteins were transferred onto a nitrocellulose membrane using a vertical blotting system for 1 h at 100 V. Primary antibodies in 1% BSA or nonfat dry milk were added to the membrane for 1 h at room temperature or were left overnight at 4 °C. The second antibody (in Tween 20 Tris-buffered saline buffer) was added for 30 min to 1 h at room temperature. Antibodies were used as follows: PARP (46D11) rabbit mAb (Invitrogen), 1:1,000 in milk; caspase-8 (1C12) mouse mAb (Invitrogen), 1:1,000 in BSA; GAPDH rabbit, 1:1,000 in milk or 5% BSA (Invitrogen), 1:3,000 2nd Ab rabbit (GE Healthcare); 1:10,000 2nd Ab mouse (Jackson ImmunoResearch Laboratories, Newmarket, UK).

##### LC/MS

Peptide mixtures were analyzed using nanoflow liquid chromatography (LC-MS/MS) on an EASY-nLC system (Proxeon Biosystems, Odense, Denmark (now Thermo Fisher Scientific)) on-line coupled to an LTQ Orbitrap XL or LTQ Orbitrap Velos instrument (Thermo Fisher Scientific, Bremen, Germany (29, 30)) through a nanoelectrospray ion source (Proxeon). Approximately 4 μg of the peptide samples in 5 μl were directly loaded onto a 15-cm column with 75-μl inner diameter, packed in-house with 3-μm reversed-phase beads (ReproSil-Pur C18-AQ, Dr. Maisch, Ammerbuch-Entringen, Germany, GmbH). Peptides were separated and directly electrosprayed into the mass spectrometer using a 145 min method including a linear gradient from 5% to 30% acetonitrile in 0.5% acetic acid over 93 to 97 min at a constant flow of 250 nl/min.

The LTQ Orbitrap XL and LTQ Orbitrap Velos were operated in data-dependent mode, switching automatically between full-scan MS and MS/MS acquisition. Instrument control was through Tune 2.6.0. and Xcalibur 2.1.0. Full-scan MS spectra (*m*/*z* 300–1,650) were acquired in the Orbitrap analyzer after accumulation to a target value of 10^6^ in the linear ion trap. Spectra were acquired with a resolution of 60,000 at 400 *m*/*z*. The 5 (LTQ Orbitrap XL) and 15 (LTQ Orbitrap Velos) most intense ions with charge states ≥ +2 were sequentially isolated with a target value of 5,000 and fragmented using collision-induced dissociation in the linear ion trap with a normalized collision energy of 35%. The activation q was set at 0.25, and the activation time was set at 30 ms and 10 ms for the LTQ Orbitrap and LTQ Orbitrap Velos, respectively. The ion selection threshold was set at 500 counts for collision-induced dissociation MS/MS. Maximum ion accumulation times of 1,000 ms and 500 ms for full scans and 150 ms and 25 ms for collision-induced dissociation MS/MS scans were set for the LTQ Orbitrap XL and the LTQ Orbitrap Velos, respectively. The dynamic exclusion was 90 s with early expiration enabled (count: 2; S/N threshold: 2). Standard MS parameters were set for all experiments as follows: 2.2 kV spray voltage; no sheath and auxiliary gas; 200 °C heated capillary temperature; predicted and normal automatic gain control enabled for Velos analyses (for Orbitrap data, normal automatic gain control was enabled); 110 V tube lens voltage (LTQ Orbitrap) and 50% to 60% S-lens radio frequency level (LTQ Orbitrap Velos); if used, a lock mass of *m*/*z* 445.120024 was applied (29); for LTQ Orbitrap Velos measurements, the lock mass abundance was set at 0%.

##### Sample Processing

For the experiments DOUBLE E1 R0K0, DOUBLE E2 R0K0, and DOUBLE E2 R10K8, Jurkat T cells were labeled in both heavy and light SILAC medium. Amino acids marked in the experiment title indicate the labeling of the TRAIL-induced cells. Reverse labeling was performed as well. For triple-labeling experiments (TRIPLE Inh TRAIL M1–3), light cultures were treated with mock, heavy cultures were treated with TRAIL, and medium cultures were treated with both z-VAD-FMK inhibitor and TRAIL.

##### Data Analysis

Raw files of each double- and triple-labeling Jurkat T cell experiment were analyzed together using the in-house-built software MaxQuant (version 1.1.1.35 (31, 32)). Each raw file from a particular slice was defined as a separate experiment in the experimental design file to obtain peptide ratios for each peptide in each slice. The derived peak list was searched with Andromeda (33) against the human International Protein Index protein sequence database (ipi.HUMAN.v3.68.fasta; 87,083 entries) supplemented with 262 frequently observed contaminants such as human keratins, bovine serum proteins, and proteases and concatenated with the reversed copies of all sequences. We required strict enzyme specificity with cleavage C-terminal after K, R, or D (trypsin + Asp-C), allowing up to two missed cleavage sites. Fixed modifications of cysteine carbamidomethylation (Cys 57.021464 Da) and variable modifications for the N-acetylation of proteins (N-term 42.010565 Da) and the oxidation of methionine (Met 15.994915 Da) were specified. Double or triple labeling was defined accordingly. The minimum peptide length was set as six amino acids. Scoring was performed in MaxQuant as described previously (31). Parent masses and fragment ions were searched with initial mass tolerances of 7 ppm and 0.5 Da, respectively. False discovery rates (FDRs) at the peptide and protein levels were fixed at 1%, including automatic filtering on peptide length, mass error precision estimates, and peptide scores of all forward and reversed peptide identifications. The re-quantification feature was enabled. Reported protein groups had to be identified by at least one “razor peptide” (a peptide most likely belonging to the protein group) in order to be accepted. Quantitation was based on unique and razor peptides only, and a minimum of two ratio counts was required. Complete protein and peptide lists, as well as the underlying RAW files, are available in the Proteome Commons Tranche database.

##### Statistical Identification of Cleavage Events

The obtained lists of peptides and proteins were further processed with an in-house-developed program implemented in MATLAB (MathWorks, Natick, MA). All ratios were converted into log2 values. Ratios of reverse labeling experiments were inverted beforehand. A protein was determined to be an apoptosis substrate if the peptides in the slices representing molecular weights smaller than that of the full-length protein were more abundant in apoptotic cells than in untreated cells. A statistical non-parametric test was applied to determine the confidence of the identification of cleaved proteins. In brief, for each identified protein group, the slice with the most peptides that had SILAC ratios smaller than 1 between TRAIL treated cells and untreated controls was selected as slice s_0_ containing the uncleaved protein. The *t* test statistics were calculated between the ratios of peptides in slice s_0_ and ratios of peptides in slices s < s_0_, for which the average ratio was larger than 1.5. The statistical test was repeated 1,000 times with randomly permutated slices to evaluate the FDR. Finally, this value was corrected for multiple-hypotheses testing via the Benjamini–Hochberg method (34).

For each protein group, a plot presenting the identified peptides as boxes in a two-dimensional map was generated. The *x*-axis of the map represented the position of the peptide with respect to the protein sequence. The *y*-axis represented the slice in which the peptide was detected. The boxes were color-coded according to the ratio between TRAIL-treated and untreated cells. If peptides cleaved at the C-terminus of aspartic acid were identified, the corresponding cleavage site was marked by a solid line if the SILAC ratio of the cleaved peptide was greater than 1.5, and by a dotted line otherwise. Similarly, potential cleavage positions that matched the sequence motif x[ET]xD and known cleavage positions were marked in the plots as additional information for the reader. Protein sequences and domains were obtained from Uniprot web services.

The second *y*-axis in the plots represented the estimated molecular weight. The mapping of slices to molecular weights was done via linear regression. For each protein group, the intensity-weighted average of slices, $s_{i}=\frac{\sum_{k=1}^{K}k{/}_{ik}}{\sum_{k=1}^{K}{/}_{ik}}$, was determined, where *k* = 1, …, *K* enumerate the slices and *I_ik_* is the extracted ion current of protein *i* in slice *k*. The linear function ln(*m*) = *a* + *bs* with the parameters *a* and *b* was then fitted to the data (*s_i_*, ln(*m_i_*)), where *m_i_* is the molecular weight of protein *i*.

The MaxQuant results, the results of the filtered substrates including statistical information, and the list of known cleavage sites were uploaded to the publicly available database MaxQB (35). Additionally, the three-dimensional cleavage plots of all identified cleavage substrates have been visualized in MaxQB can be obtained at http://maxqb.biochem.mpg.de. Instructions on how to use MaxQB for visualizing and extracting cleavage information are provided as supplemental information. In addition, all .m files for the MATLAB script are provided on the MaxQB web page to allow researchers to analyze their data with regard to proteolytic cleavage events (http://maxqb.biochem.mpg.de/mxdb/project/show/9213156110000).

##### Data Representation

Data were depicted using GraphPad Prism (version 5.04) and the free software environment R.

##### Enrichment Analyses

Cleavage events are more likely to be detected in highly abundant proteins. In order to remove this effect of abundance as a confounding variable, we first generated a background distribution of the whole proteome data with an intensity distribution identical to that of the cleaved proteins. To this end, the intensity range was binned and each protein group from the whole dataset was randomly drawn with a probability equal to the proportion of cleaved proteins in the corresponding bin out of the total number of cleaved proteins. Fisher's exact test was then used to identify gene ontology categories that were significantly enriched or depleted (Benjamini–Hochberg FDR = 0.02) in the cleaved population relative to the background population.

## RESULTS AND DISCUSSION

### 

#### 

##### Development of a SILAC-based Quantitative Approach to Detect Proteolytic Substrates

**Fig. 1.**
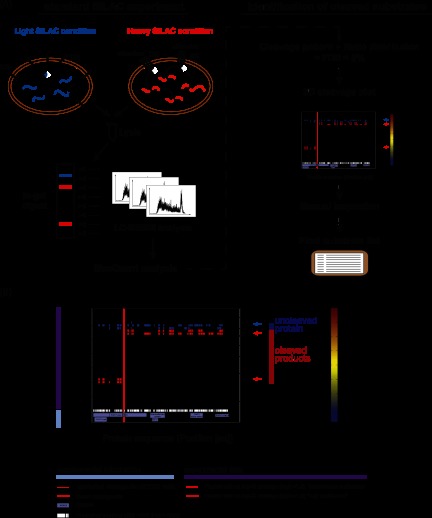
**Representation of the SILAC-based approach for the detection of proteolytic substrates.** *A*, schematic overview of the SILAC experiment followed by stringent statistical data filtering to extract caspase-dependent cleavage substrates. In a SILAC double-labeling experiment, the cells are treated with an apoptotic stimulus (heavy condition) or mock (light condition). Cells are combined and lysed, and proteins are separated via SDS-PAGE. After in-gel digestion, each slice is analyzed via LC-MS/MS. Cleavage candidates are identified on the basis of specific cleavage criteria and via the application of statistics to the data. Graphs are generated for each candidate and manually inspected before the generation of a final substrate list. DR, death receptor. *B*, Three-dimensional cleavage plot representation. The plot combines both experimental data and background information. Detected peptides are depicted as squares defined by slice and sequence localization, including the color-coded corresponding ratio. Detected cleavages C-terminal of Asp are highlighted within the plot by red lines. Supplemental information includes theoretical cleavage of the protein (peptides are plotted as squares), known and hypothetical cleavage sites along the protein sequence, and known protein domains and motifs. Blue and red arrows indicate the location of the uncleaved and cleaved regions, respectively. Information provided within the graph is explained below the three-dimensional cleavage plot. Experimental data are depicted in the main upper part of the plot, and specific information at the bottom of the plot.

When proteolytically cleaved proteins change their molecular weight relative to their uncleaved counterparts, it can be detected in an SDS gel as a shift of the cleaved protein to a lower-molecular-weight region. We took advantage of this fact to extract information about cleaved proteins from complete proteome datasets. For direct comparison, we combined treated and untreated states in one quantitative SILAC experiment. As both samples were merged at the cell level, this eliminated errors arising from a lack of reproducibility and differences in sample processing. In our SILAC experiments, untreated cells were labeled with light RPMI medium (Arg0; Lys0) and cells used for treatment were labeled with heavy medium (Arg10; Lys8) before the induction of apoptosis (Fig. 1*A*). We also included label-swap experiments as an additional check of the method (supplemental Fig. S1*A*).

We applied the SILAC-based approach to investigate cleavages induced by treatment with TRAIL. Populations of SILAC-labeled Jurkat T cells that had been treated with TRAIL or mock control for 5 h were merged, and their lysates were separated on one-dimensional SDS gels. To achieve high molecular weight resolution, we cut the gel into between 28 and 36 horizontal slices and followed this with standard in-gel digestion and LC-MS/MS analysis on ion trap Orbitrap instruments. Data were analyzed with the MaxQuant software as in our standard workflows; however, the output data were further processed in order to identify cleaved caspase substrates (see “Experimental Procedures”) by means of sophisticated filtering. For this processing step, we developed a statistical algorithm that specifically extracts proteins with a distinct peptide distribution pattern as a function of gel slice position. In-gel digestion of a particular gel slice creates peptides from the embedded proteins that represent a certain mass range (the apparent molecular weight of that gel position). The software provides the localization of all detected peptides from each protein in two dimensions: within the gel according to the apparent molecular weight region, and along the protein sequence according to the location of the identified peptide (Fig. 1*B*). Peptides from the uncleaved protein are expected to be located in higher molecular weight regions and to cover the protein sequence without bias to sequence location. In contrast, cleaved apoptotic fragments of the same protein should migrate to a lower molecular weight region, and the identifying peptides should span only a certain part of the protein sequence in accordance with the cleavage position. The quantitative information obtained via SILAC labeling then provides a third dimension. These SILAC peptide ratios encode information about the extent to which the protein or fragment comes from the treated or untreated state. Peptides from the uncleaved protein are mainly derived from the untreated cells (negative treated/untreated ratio after log transformation) when they are from a high mass region, whereas peptides derived from corresponding cleaved fragments are mainly derived from the apoptotic state (positive treated/untreated ratio) if they are from the relatively lower gel position. The slice with the highest sequence coverage combined with a median log ratio around zero corresponds to the uncleaved protein. Slices at lower molecular weights with median positive ratios correspond to the cleaved fragments. The third dimension is represented in our plots as a heat map value (red for positive SILAC log ratios and blue for negative log SILAC ratios; Fig. 1*B*). Importantly, the above-mentioned criteria should be fulfilled concurrently; that is, the uncleaved protein and its SILAC and sequence values should be consistent with the cleaved product or products and their location, SILAC ratios, and distributions of peptides in the protein sequence.

To statistically formalize these criteria, the software defines these cleaved and uncleaved regions of a protein and calculates *p* values and an FDR for the significance of being a cleavage product (see “Experimental Procedures”). Only proteins with an FDR value below 5% were considered as significant candidates, and only for these were graphs created. We marked theoretically detectable peptides to give an impression of the highest possible sequence coverage of the protein. In addition to the experimental data, we also included data from the literature in the graphs. For instance, known cleavage sites of proteins in the substrate database that we created (see below), as well as possible cleavage sites following the x[ET]xD caspase cleavage motif, are indicated as red lines. Known domains of the protein derived from Uniprot are depicted as small blue boxes. In addition, we marked the specific protein position with a red line where peptides showed cleavage according to enzymatic digestion with Asp C. This is because caspase cleavage followed by tryptic digestion creates semitryptic peptides in shotgun proteomics experiments that, if detected, can mark the exact site of caspase cleavage. For these peptides, we distinguished cleavages with high confidence (ratio [log2] > 1.5) and intermediate confidence (ratio [log2] < 1.5); these values were chosen based on the distribution of known caspase cleavage products. For high-confidence cleavages, the explicit cleavage site is denoted in the output table, as well as the sequence window of ±3 amino acids surrounding the cleavage site. In addition, we calculated molecular weight distributions that relate the position on the one-dimensional SDS gel to the molecular weight of the proteins detected in the entire proteome dataset of this experiment. This relationship is depicted in supplemental Fig. S1*B* and is used to provide a molecular weight scale on the right-hand *y*-axis of each plot. We further manually inspected the automatically filtered candidates for consistency across all six experiments (see below) and verified them for inclusion in the final substrate list.

##### Identification and Characterization of TRAIL-induced Proteolytic Substrates

**Fig. 2.**
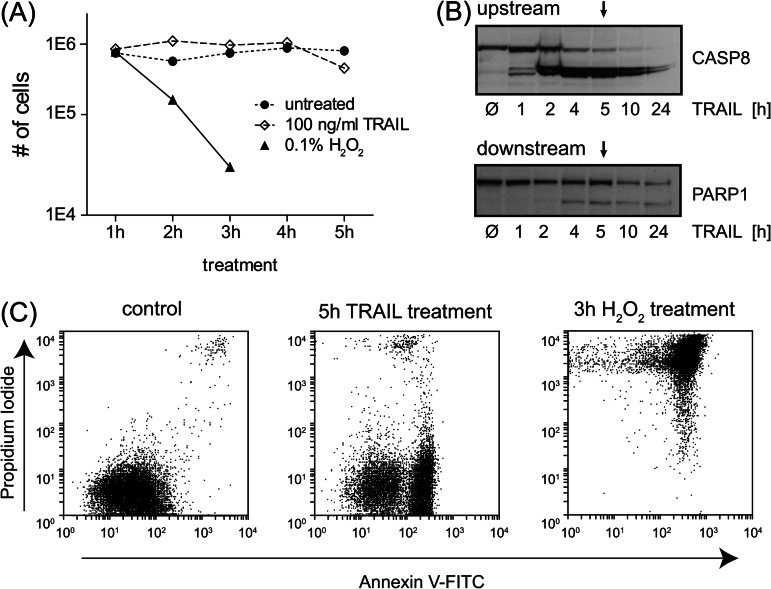
**Apoptosis verification after TRAIL induction.** *A*, viability study after induction of apoptosis or necrosis compared with mock treatment. Cells were treated with 0.1% H_2_O_2_ and 100 ng/ml TRAIL or mock for several hours. The permeability of the cells was checked with a cell counter; cell numbers are plotted. *B*, Western blot analysis of apoptotic markers. Cleavage of the upstream substrate CASP8 and the downstream substrate PARP1 was detected after TRAIL treatment within 24 h. Arrows point to the treatment at 5 h, the time point that was used for the SILAC experiments. *C*, flow cytometry analysis distinguishing between apoptosis and necrosis. Experiments show a response to propidium iodide and Annexin V-FITC in mock, TRAIL, and H_2_O_2_ treatments. TRAIL-treated cells revealed a clear response to Annexin V after 5 h, but no necrotic effects were detected.

Because no global whole proteome studies had been reported on the specific proteolytic substrate spectrum of TRAIL-induced apoptosis, we treated Jurkat T cells with different concentrations of TRAIL and monitored their state over time via light microscopy (see “Experimental Procedures”). Based on the results, we chose a TRAIL concentration of 100 ng/ml and stimulation for 5 h. Microscopic, flow cytometric, and immunoblotting results (Figs. 2*A*–2*C*) showed that this time point optimally covers both upstream and downstream apoptotic events (initiating caspases and substrates downstream of caspases, respectively), which should allow us to cover a broad spectrum of apoptotic cleavage events. In addition, the results of our H_2_O_2_ treatment—an unspecific cytotoxic agent—show that we clearly separated apoptosis from uncontrolled necrotic cell death (Figs. 2*A* and 2*C*).

Next, we applied the above-mentioned conditions in a SILAC experiment treating Jurkat T cells for 5 h with TRAIL (100 ng/ml) and mock. We performed three biological double-labeling experiments and three biological triple-labeling experiments comparing TRAIL-treated and mock-treated states. In the triple-labeling experiments, we used only the light and heavy channels; the medium condition was additionally inhibitor treated, as mentioned below.

For substrate identification, we combined all six experiments. When we identified a candidate as significant in one of the experiments (FDR < 5%), the software created a three-dimensional cleavage plot for the other experiments (even if they were not significant) to allow better comparison among the different experiments. We derived significances for each plot from the FDR columns of the output of our script and counted how often each protein had been identified as statistically significant in the six experiments. In the final manual validation, only candidates with an appropriate peptide distribution pattern that were found to be significant at least three times were designated as TRAIL-induced apoptosis substrates and considered for further analyses (supplemental Table S1).

**Fig. 3.**
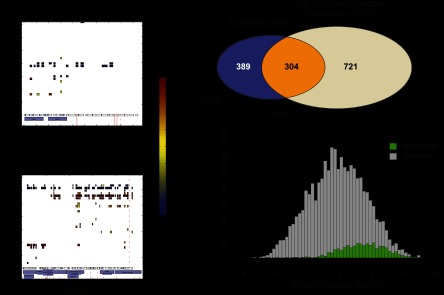
**Identification of TRAIL-induced cleaved substrates.** *A*, positive markers of apoptotic cleavage. Three-dimensional cleavage plots are depicted for the substrates CASP8 and PARP1. For both substrates, strong negative ratios were identified in the uncleaved region, whereas cleaved fragments showed high positive ratios indicating strong cleavage. *B*, identification of known and novel substrates. Substrates detected in our dataset were compared with a list of known substrates. Slightly more than half of the substrates were novel cleavage substrates induced by TRAIL treatment. *C*, comparison of the whole proteome and the identified substrates. The intensity distribution of all proteins is depicted, with the population of cleaved substrates highlighted in green. Cleavage substrates span the higher intensity region of the complete detected proteome.

This procedure identified a plethora of positive controls known to be proteolytically cleaved during apoptosis, prominent examples of which are presented in Fig 3*A*. CASP8 shows a clear uncleaved region with good sequence coverage. The N-terminal cleaved fragments in the range of 43 kDa and 24 kDa corresponding to the most probable cleavage at D374 and D216 are present (4). These two cleavage sites correspond to the first and second cleavage of CASP8, for which another cleavage at D384 is known. For the well-studied downstream substrate PARP1, we covered nearly the complete sequence in the uncleaved region (113 kDa) with highly negative ratios (indicating absence of the protein in the treated state and therefore almost complete cleavage). In addition, we also obtained high sequence coverage of the fragments, and the mass of the fragments fit with their sequence region of the protein (89 kDa and 24 kDa). The region of the cleavage site is clearly visible and is in agreement with a known cleavage site on PARP1. This site, DEVD[214], is located directly within the DNA-binding region. Through cleavage of the N-terminal part of the protein, PARP1 ceases to be recruited to the DNA and consequently loses its catalytic activity in DNA damage repair. We also detected Caspase-3, Caspase-6, and Caspase-2, and many other well-documented caspase substrates of both early and late cleavage events such as BID, DFFA, PAK2, VIM, LMNB1/B2, and ROCK1 (supplemental Table S1). BID mediates the crosstalk between the extrinsic and intrinsic form of cell death via accumulation of its 15-kDa fragment tBid at mitochondria, initiating mitochondrial outer membrane permeabilization (36). DFFA (ICAD) is an inhibitor of the DNase CAD, which is inactivated by cleavage. Cleavage of this inhibitor then allows free CAD to translocate to the DNA and degrade chromosomes into nucleosomal fragments, a characteristic feature of apoptosis.

After statistical filtering and manual inspection, we obtained 693 cleaved substrates in response to TRAIL-induced apoptosis (Fig. 3*B*; supplemental Fig. S2*A*). To generate a list of known substrates for comparison, we accessed the caspase substrate database CASBAH (37) and data from two large-scale investigations (11, 23). This analysis showed that 304 of our substrates were already known as verified or potential caspase substrates, and 389 of our candidate proteins were entirely novel. Together, these results demonstrate that our screen covered a substantial part of the known substrates of all caspase substrates while identifying a very large set of novel TRAIL-induced cleavage products. This attests to the sensitivity of our approach but also suggests that only a fraction of all cleaved substrates have been described to date (38, 39).

We next plotted the intensity distribution spanned by the complete proteome experiment in comparison with the intensity fraction covered by the statistically significant cleaved substrates (Fig. 3*C*). Cleavage substrates were distributed toward the higher abundant area of the complete proteome. This is not surprising as verification, as a substrate requires substantially more information than mere identification in the proteome. Interestingly, although our data are biased toward the more abundant part of the proteome, the substrates are relatively flatly distributed across the accessible abundance range. This shows that the tendency to be a caspase substrate is not strongly correlated with protein abundance, at least in the abundance range covered by this study.

Detected substrates span a broad molecular weight range and encompass proteins ranging from about 20 kDa to up to 200 kDa. Nevertheless, very small proteins are underrepresented within the substrate population, which might be the result of limitations of the gel-based MS approach (supplemental Figs. S2*B*, S2*C*).

**Fig. 4.**
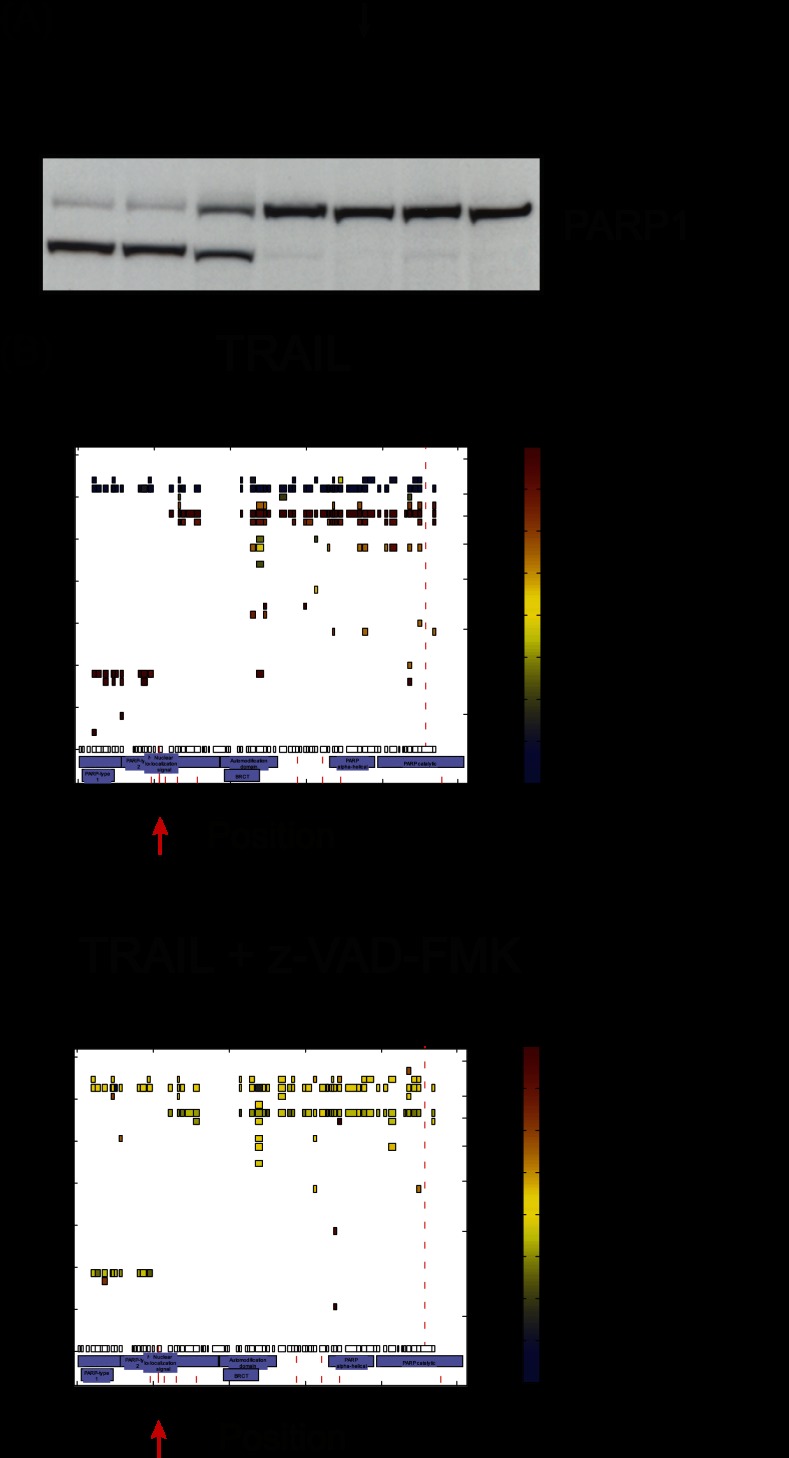
**Identification of caspase dependence of the cleaved substrates.** *A*, inhibition of apoptosis by z-VAD-FMK. Different concentrations of the pan-caspase inhibitor z-VAD-FMK were tested, and cleavage was detected via Western blot against the known caspase substrate PARP1. The arrow points to the condition used for the SILAC experiments. *B*, comparison of TRAIL-treated and inhibitor-treated samples. TRAIL treatment generated a clear cleavage pattern, as, for example, for PARP1, including characteristic ratios in the uncleaved and cleaved regions (upper panel). Inhibitor-treated samples showed no characteristic ratio pattern (lower panel). In some cases, slight background cleavage could be detected in both mock and treated samples generating peptides in the fragment slices with 1:1 ratios. Red arrows highlight the localization of the known cleavage site.

Proteolytic substrates are not necessarily the result of caspase cleavage; they also can be cleaved by other proteases, which could be either activated by or independent of upstream caspase cleavage. To verify that the cleaved substrates were truly caspase dependent, we incubated Jurkat T cells with the pan-caspase inhibitor z-VAD-FMK before treatment with TRAIL. We determined the best inhibitor conditions by using different concentrations of z-VAD-FMK and the known caspase substrate PARP1 (Fig. 4*A*). Based on these results, we utilized a triple-SILAC approach treating SILAC-labeled cells with mock (light SILAC condition), TRAIL (5 h; heavy SILAC condition), or TRAIL (5 h) and z-VAD-FMK at 50 μm (medium SILAC condition). We performed three biological replicate experiments and analyzed the samples as described under “Experimental Procedures.” TRAIL-treated samples (heavy/light ratio) showed clear cleavage patterns (Fig. 4*B*, upper panel), whereas inhibitor-treated samples showed no peptide ratio difference relative to the mock-treated population (Fig. 4*B*, lower panel) (medium/light ratio). Note that peptides of caspase-dependent substrates such as PARP1 could also be found in molecular weight regions of the cleavage fragments; however, these have one-to-one SILAC ratios (0 in log2) between inhibitor-treated and control conditions, showing that they originate from background (TRAIL-independent) cleavage. Because all significant cleavage events were abolished by the inhibitor, we conclude that they all were caspase dependent.

##### Identification of Proteolytic Cleavage Sites

**Fig. 5.**
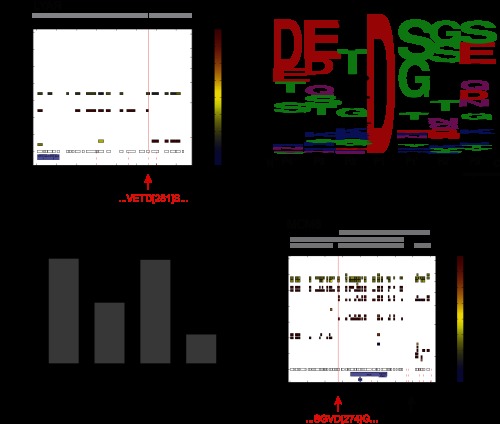
**Localization of cleavage sites.** *A*, detection of explicit cleavage sites. As a representative example of explicit cleavage site detection, the novel substrate LYAR is depicted, for which an explicit cleavage was detected at D281. The peptide cleaved C-terminally of Asp was identified with high confidence and matched with a hypothetical cleavage site, whereas the uncleaved peptide was identified in the slice containing the uncleaved protein with a negative ratio. *B*, evaluation of caspase specificity via sequence logo representation. We extracted the sequence windows (Asp ± 3aa) of all explicit cleavage sites and generated a sequence logo for the substrates. The output data matched with current knowledge and supported the preference for small amino acids at position P1′. *C*, representation of categories providing information on cleaved regions. Besides explicit cleavage sites, cleaved regions could be narrowed down to small regions along the protein sequence. We defined four different categories describing the information obtained about the cleavage position. *D*, multiple cleavages by caspases. In several cases, multiple cleavage events could be located within one protein. Protein MCM6, for example, was cleaved at the same time either once or twice along its protein sequence, generating five different cleavage fragments. The explicit cleavage site D274 is indicated by a red arrow; the black arrow highlights the approximate second cleavage site.

In addition to identifying cleavage substrates of caspases, our approach might allow the identification of specific caspase cleavage sites in a subset of cases. To test this, we searched for peptides with either an N- or a C-terminal caspase cleavage site and a tryptic site on the other terminus. We also required that the peptide is located in the region indicated by our statistical algorithm as the probable cleaved region (see “Experimental Procedures”). Using this approach, we were indeed able to identify 93 explicit cleavage events in 86 proteins, including known ones that served as positive controls and novel ones. These sites, including the sequence windows of ±3 amino acids, are listed in supplemental Table S3. Several novel potential substrates such as the cell-growth-regulating nucleolar protein LYAR also were found in this way (Fig. 5*A*). For this protein, we mapped the cleavage site to the position D281 overlapping with the hypothetical cleavage motif x[ET]xD. A sequence logo analysis (Fig. 5*B*) supported earlier findings on intrinsically induced apoptosis that characterized cleavage motifs as diverse (6). Cleavage is common at an aspartate C-terminal to a small amino acid such as serine, glycine, or alanine, as reported previously (6, 11). We concluded from this that TRAIL-induced cleavage patterns are similar to those observed in intrinsic caspase-mediated events.

In cases when the caspase-cleaved peptide was not confidently identified, the cleavage event was often still mappable to a particular region of the protein. In 159 proteins, the cleavage was mapped to a region of about 100 amino acids (Fig. 5*C*; supplemental Table S3). In 50 of these cases, the cleavage area overlapped with only one x[ET]xD motif (*implicit sites*). In 85 proteins, there was more than one motif in the cleavage area (*area*). In the remaining cases, a different cleavage motif was presumably used by the responsible protease.

In several cases, we determined multiple cleavage events within the same protein (Fig. 5*D*). We found, for instance, two cleavage events in the DNA replication licensing factor MCM6, one of which was already known and could be mapped directly as an explicit site (D274). For the second cleavage, two possible motifs matched within the C-terminal region close to position 770. From the positions of the fragments in the gel, we determined that either or both cleavages can occur, indicating that there appears to be no preference for one of them as an initiator site (Fig. 5*D*). As either cleavage presumably inactivates the protein, such a cleavage pattern is functionally sensible for substrate inactivation.

As these results show, our whole protein approach is not necessarily limited to the identification of cleaved proteins and in many cases might yield detailed information about the cleavage site or region. Even the identification of cleavage regions can already be of great importance for tracking the effect of the cleavage by providing information about cleaved domains or motifs, for instance.

##### Global Analysis of Detected Proteolytic Events

**Fig. 6.**
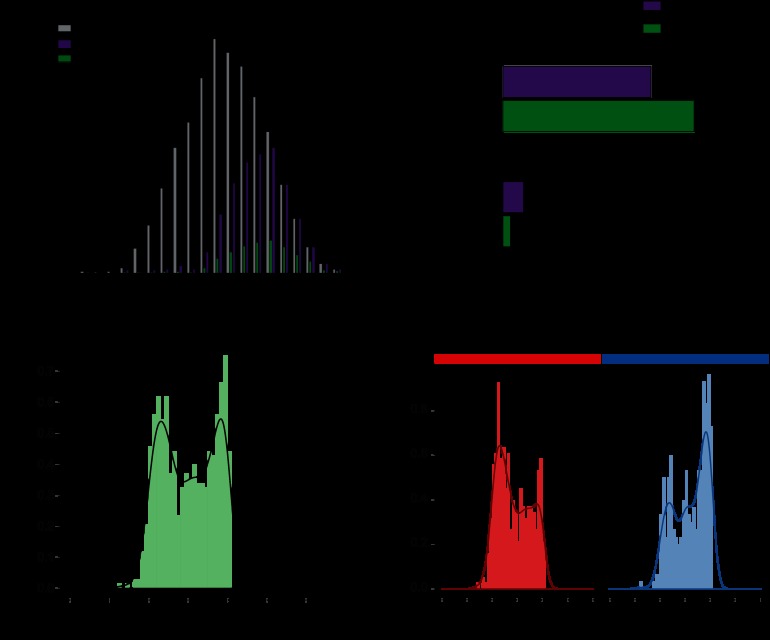
**Global analysis of cleaved substrates.** *A*, calculation of a background population for enrichment testing. To perform unbiased enrichment testing of cleavage substrates *versus* the whole proteome, a background population was extracted from the whole proteome data spanning intensity ranges equal to those of the substrate population. All three categories are depicted. *B*, enrichment analysis of substrates. Proteins in complexes are slightly enriched within the substrate population. Proteins assigned to mitochondrion are significantly de-enriched within the substrate population. Bg, background. *C*, distribution of uncleaved ratios of all substrates. Median ratios of the uncleaved protein regions follow a clear bimodal distribution in all experiments. *D*, representation of uncleaved protein ratios with regard to proteins in complexes. Substrates annotated in CORUM showed clear tendencies toward the right-hand peak; substrates not assigned to complexes tended toward stronger negative ratios.

Bioinformatic analysis of our substrate proteome revealed that it covered a broad range of different pathways with known and novel substrates showing similar trends (supplemental Table S4). In particular, many substrates belong to RNA-dependent pathways, endocytosis, spliceosomal, and cell-cycle-related processes. We next extracted protein populations enriched or depleted in our substrate population relative to the complete detected proteome using Fisher's exact test (Benjamini–Hochberg FDR = 0.02). Because the substrate population is biased toward higher protein expression, as determined earlier (Fig. 3*C*), we first created a background protein population spanning the same intensity region as the cleaved substrates (Fig. 6*A*) to serve as a reference population. Interestingly, the substrates showed an asymmetric distribution between depleted and enriched categories (supplemental Table S5). TRAIL-induced apoptosis appears to target a broad range of functions without strong preferences, whereas a small number of pathways and compartments were clearly selected against. The latter encompassed the endoplasmic reticulum, mitochondria, and inner membrane proteins in general, as discussed below.

RNA-regulated processes including RNA helicase activity and RNA splicing in particular, but also pathways such as endocytosis (CLTA, AP2A1, AP2A2, DNM2) (40), were significantly enriched in cleaved substrates. Nuclear proteins were slightly but statistically significantly enriched, as were proteins in complexes (derived from the CORUM database (41)) (Fig. 6*B*). In contrast, proteins intrinsic to membrane were significantly underrepresented, and proteins in mitochondria and endoplasmic reticulum were underrepresented by more than 200% within the cleaved substrate fraction (Fig. 6*B*). Proteins in endoplasmic reticulum and mitochondria might not be accessible to caspases, or they might not contain as many cleavage sites (cleavage motifs) because they have to fulfill important tasks in controlled cell death. In any case, this is an interesting observation given that mitochondria dynamics and fission are known apoptotic effects (42). Regardless of the mechanism, our data indicate that the mitochondria are protected from caspase cleavage, which appears reasonable given the central function of mitochondria in intrinsically induced—but also extrinsically caused—apoptosis. In contrast, nuclear pore proteins are preferentially cleaved by caspases, and nuclear proteins are enriched as substrates, which is in agreement with the fact that nuclear breakdown is a hallmark of apoptosis.

The preceding analysis was focused on the identity of the substrate proteins only. In a next step, by evaluating the SILAC ratios of the apoptotic substrates at the gel positions of the full-length proteins (uncleaved ratios), we noticed that they displayed in all experiments a bimodal distribution (Fig. 6*C*). Classical upstream (*e.g.* caspases) and downstream substrates (*e.g.* PARP1, LMNB2, ROCK1) were observed with strong negative ratios, indicating that a large percentage of these substrates had been cleaved at this time point (5 h). In contrast, proteins in the right-hand peak had ratios around zero, indicating that most of these proteins remained uncleaved, even though fragments with clear positive ratios marked them as apoptotic substrates. Splitting the population into novel and known substrates showed a clear tendency of novel substrates toward the right-hand distribution (low percentage of substrate cleavage; supplemental Fig. S3*A*). This might reflect the sensitivity of our approach, as proteins cleaved with lower stoichiometry are more difficult to detect. Bioinformatic analysis of the enrichment of protein classes within the two different peaks with a Fisher's exact test (Benjamini–Hochberg FDR = 0.02) revealed that proteins in complexes were highly enriched in the right-hand peak (Fig. 6*D*; supplemental Fig. S3*B*). Thus complex members often appear to be cleaved with less efficiency than proteins not involved in complexes.

##### Caspase Cleavage of the Condensin Complex

To perform a physical and functional interaction analysis of our substrate population, we used the STRING database (STRING 9.0; highest confidence (score 0.90)) and visualized and analyzed results in Cytoscape (version 2.8.2). We grouped the substrates into known and novel proteins (green and red, respectively, in supplemental Fig. S4). The center of the interaction network was formed by Caspase-3 surrounded by many known substrates such as CASP6, BID, LMNB1, PAK2, and ROCK1. Novel substrates created additional links between known cleavage substrates but also spanned further networks at the periphery of the graph. Protein clusters highlighted interactions of ribosomal and nuclear pore proteins, as well as of proteins involved in protein biosynthesis, DNA replication, transcription, vesicle transport, and endocytosis, defined by both known and novel substrates. We also noticed entire macromolecular complexes, among which proteasomal proteins and the condensin I complex were prominent. The latter is of special interest because it plays a crucial role in the formation of structurally stable mitotic chromosomes and their segregation, as well as in gene regulation and DNA repair (43, 44). The pentameric condensin I complex is highly conserved and ubiquitously found among eukaryotes. It consists of two structural maintenance of chromosome ATPase subunits and three auxiliary subunits. Only one component of the complex, the kleisin subunit NCAPH, has been reported to be cleaved upon apoptosis induction to date. This cleavage was thought to be responsible for the loss of the condensin I complex in apoptosis, contributing to a loss of chromosome structure and chromosome susceptibility to DNA fragmentation induced by a caspase-activated DNase (45).

**Fig. 7.**
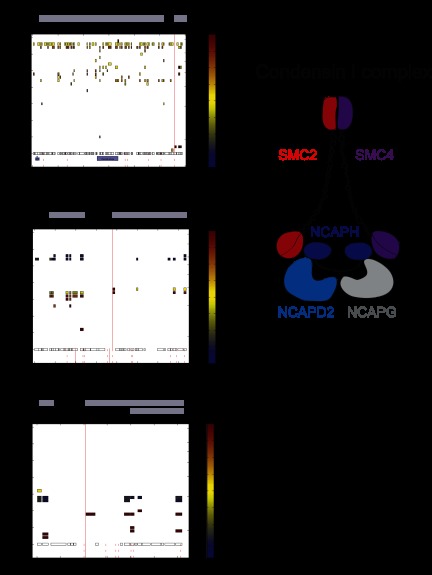
**Investigation of specific cleavage substrates in a biological context.** *A*, cleavage of the condensin I complex members. All components of the condensin I complex were identified as cleavage substrates. For the novel substrate SMC2, an explicit cleavage site was identified within the C-terminal Walker B motif (upper panel) at D1116. The known cleavage substrate NCAPH was strongly cleaved in the middle of the protein sequence, but the detected explicit cleavage site at D380 did not exactly match the literature (lower panel). *B*, identification of the autophagic protein ATG3 as a caspase substrate. We mapped an explicit cleavage site to D104 within the ATG3 sequence. Cleavage fragments indicate an additional cleavage within the C-terminal fragment.

Interestingly, we identified not only NCAPH but also all other components of the complex as cleaved substrates after TRAIL induction (Fig. 7*A*; supplemental Fig. S5). To better understand the process of condensin I complex cleavage, we extracted the three-dimensional cleavage plots of all of its members. The known substrate NCAPH was strongly cleaved in the middle of the protein, as already described in the literature. However, our detected cleavage site (D380), which matches with a potential cleavage motif, does not overlap with the known cleavage site at D366. The novel substrates of this complex SMC2, SMC4, NCAPD2, and NCAPG showed cleavage at the N- or C-termini, resulting in a slight shift of the uncleaved protein. These substrates were cleaved with less efficiency than the known substrate NCAPH. Both structural maintenance of chromosome ATPase subunits (SMC2 and SMC4) have nucleotide-binding domains at their N- and C-terminus, termed Walker A and B motifs. Within each protein, these domains interact with each other and form so-called head domains. We detected cleavages in both proteins at their N- and C-termini. Interestingly, for SMC2 we could even map an explicit cleavage site at the C-terminal part of the protein located exactly within the Walker B motif at D1116. This is remarkable because ATP-binding pockets should be difficult to access. For NCAPD2 and NCAPG, we also detected cleavage at the N- and C-terminus, respectively. These cleavage sites appear to overlap with important intra- and intermolecular interaction sites of the other proteins of the complex. Interestingly, NCAPD3 from the related condensin II complex was cleaved as well. However, in this case we located this cleavage site at position D529 in the middle part of the sequence, rather than the termini. This might indicate different modes of association of NCAPD2 and NCAPD3 with their corresponding kleisin subunits.

A recent paper investigated the function of yeast condensin by introducing several TEV cleavage sites into Brn1 (NCAPH homologue) (46). Interestingly, cleavage of NCAPH leads to opening of the condensin ring, but without releasing subunits of the complex. Intriguingly, after cleavage of NCAPH, the complex is still associated with the chromosome if the two NCAPH fragments are held together by interactions with NCAPD2, at least *in vitro*. After additional cleavage of NCAPD2, condensin is released from the chromosomes. Also, in the case of SMC2 coiled coil artificial cleavage of both strands was necessary for inactivation. In light of these findings and our data, we suggest that simple cleavage of NCAPH by caspases might not be sufficient to release condensin from the chromosome. Additional proteolytic cleavage events in other subunits—preferentially at the interaction sites—might be necessary in order to fully inactivate the complex and disengage its components. Sites in condensin I complex subunits other than NCAPH were observed with lower SILAC fold changes, suggesting that the specific location of cleavage is less important than the fact that a second cleavage occurs. Although these mechanisms are speculative, the cleavage sites identified here are mechanistically plausible and may serve as starting points for further functional dissection of the cleavage of the complex.

##### Caspase Cleavages of the Proteasome

Recently, several groups have suggested that protein degradation by caspases and by the proteasome are interlinked by the reciprocal cleavage of some of its components, and several proteasomal subunits have been identified as apoptosis substrates (6). Here we identified cleavage events in the 20S core particle and in the 19S regulatory particles, covering both base and lid proteins (47, 48) (supplemental Fig. S6). As with the condensin complex, stoichiometries of substrate cleavages were relatively low. From the regulatory particles, we found the previously known substrate subunits Rpn2, Rpn3, Rpn10, Rpt1, Rpt5, Rpt6, and Rpt4, as well as the novel substrates Rpn5 and Rpt2 (6). From the core particle, we identified PSMA5 and also observed cleavage of the 11S regulatory cap, PA28γ. These findings support the hypothesis of a negative feedback loop in which caspases and the proteasome are interlinked in the process of apoptosis (49). In healthy cells, caspases as the effectors of apoptosis are tightly regulated by the proteasome, and their protein levels are reduced via proteasomal control. Upon caspase activation by apoptosis, proteins mainly within the regulatory part of the proteasome are cleaved in a caspase-dependent manner, and proteasomal degradation is inhibited. Our findings support this general picture and supply a number of specific substrates and sites in the proteasome.

An earlier study identified caspase cleavage of three proteins of the 19S regulator particle complex of the 26S proteasome. PSMC3 (Rpt5) and PSMD4 (Rpn10) most probably recognize the polyubiquitinated proteins, whereas PSMD1 (Rpn2) most likely holds together the lid and the base of the 19S regulator particle (49). Their cleavage might inactivate this process and partially detach the 19S regulator particle from the 20S core particle. Our data substantiate the cleavage sites of PSMC3 and PSMD1. For PSMD4, two possible cleavage sites were posited in an earlier study. Our results clearly identify cleavage at one of them, D258.

The SILAC ratios observed in our data are relatively low, suggesting sub-stoichiometric cleavage at different sites for each member protein of a complex. In order to compensate for this lower cleavage efficiency, caspases appear to cleave several members of the same protein complex, such as the proteasome, perhaps with a more robust inactivation effect as the cleavage of one specific protein.

##### Caspase Cleavages of the Autophagy Apparatus

The link between apoptosis and autophagy is also of special interest (see, for instance, Ref. 50). Autophagy is cytoprotective, whereas apoptosis inhibits autophagy when activated, counteracting its effects. Important points of crosstalk include the interaction of the autophagy protein Beclin-1 and the anti-apoptotic factor Bcl-2, as well as direct interactions of caspases and autophagic components. A recent study described the cleavage of the early autophagy marker ATG3 by CASP8 (51) as an important link between both pathways. The authors focused on the *in silico* derived site D169 and in an experimental follow-up defined it as the cleavage site of CASP8 leading to the inactivation of the autophagic process after TNF or TRAIL induction. Here, we also detected ATG3 cleavage after TRAIL induction in our Jurkat T cell system, and we identified a cleavage region including two possible sites, D169 being one of them (Fig. 7*B*, supplemental Table S1). Furthermore, we mapped an explicit cleavage site of ATG3 at D104. That site had also been found in another study (23), supporting our results. Based on the peptide information from the cleaved fragments, the cleavage at D104 appears to be more prominent than that at D169. Because peptides from the counterpart of the D169 cleavage are absent in the gel, we speculate that this cleavage is secondary. In any case, the detection of two cleavage events via our method illustrates its depth and its unbiased nature.

## CONCLUSIONS AND OUTLOOK

Here we have described a quantitative SILAC-based approach for the identification of proteolytically cleaved substrates and used it to investigate the events of apoptosis induced by the extrinsic stimulus TRAIL. Our approach uncovered nearly 700 cleavage substrates, a dataset that can serve as a resource for studying TRAIL-induced cleavage events for the community of cell death researchers. It also might be clinically relevant, because TRAIL is of great importance for cancer therapy research aiming to preferentially inducing apoptosis in tumor cells but not in normal cells (52, 53). In addition, our approach can be applied to any system in which proteolytic cleavage occurs and could therefore also be of interest in fields such as embryogenesis or neurodegenerative disease research.

After this study was finished, Cravatt and co-workers incorporated SILAC into their PROTOMAP approach (23) and elegantly used it to discover the requirement of priming phosphorylation events for apoptotic cleavage events (25). They investigated staurosporine-induced apoptosis, rather than extrinsic ligand-induced apoptosis. They detect about 700 cleaved substrates, which is reduced to about 500 substrates when an indication of cleavage products in lower molecular weight gel regions is required, as was the case in our study. About 300 substrates are common to both approaches, indicating that many of the same substrates are cleaved following intrinsic and extrinsic stimulation of apoptosis. Nevertheless, it is clear that neither approach reached completion and that there are a large number of apoptotic substrates still to be discovered. Already from the data acquired here, several features of the apoptotic substrate proteome have become apparent. For instance, we found that mitochondria are clearly underrepresented as cleavage substrates and that stable complexes appear to be disabled by several apoptotic cleavage events in different complex members, each with less than full stoichiometry. Our data also support the notion that apoptotic cleavage events are not randomly distributed in the cellular proteome and instead target specific proteins and pathways (39). In this regard, it is interesting to note that many of the novel substrates discovered here link to already known targets or networks. In conclusion, we note that MS-based proteomics is still improving in terms of the speed and depth of analysis and that it should soon be possible to investigate and compare different apoptotic stimuli and conditions, as well as inhibitors, providing valuable input for studying the mechanisms of apoptosis.

## Supplementary Material

Suppl Table S1

## Footnotes

^1^ The abbreviations used are:

CASP8: Caspase-8
FDR: false discovery rate
FMK: fluoromethylketone
SILAC: stable isotope labeling with amino acids in cell culture
TRAIL: tumor necrosis factor–related apoptosis-inducing ligand.
