# Supplementary material for: A SILAC-based Approach Identifies Substrates of Caspase-dependent Cleavage upon TRAIL-induced Apoptosis
Source: Mol Cell Proteomics. 2013 Jan 13;12(5):1436–50. doi: 10.1074/mcp.M112.024679 (PMC3650350; doi:10.1074/mcp.M112.024679)
Supplement: Suppl Table S1 [file supp_12_5_1436__index.html]

A SILAC-based approach identifies substrates of caspase-dependent cleavage upon TRAIL-induced apoptosis — A SILAC-based Approach Identifies Substrates of Caspase-dependent Cleavage upon TRAIL-induced Apoptosis — SILAC-based Approach to Globally Detect Protease Substrates — Suppl Table S1 

# A SILAC-based Approach Identifies Substrates of Caspase-dependent Cleavage upon TRAIL-induced Apoptosis

## Supplemental Data

**Files in this Data Supplement:**

- suppl material - Table of Content Suppl Figures S1-S6 Description of Supplemental Information Description of Suppl Tables S1 - S5
- Suppl Table S1 - Excel Table containing all substrates including 3D cleavage plots, cleavage statistics, uncleaved and cleaved ratios, sequence coverages and explicit site information.
- Suppl Table S2 - List of all known human cleavage substrates as defined within the text.
- Suppl Table S3 - Table containing substrates with cleavage site information (related to Figure 5C).
- Suppl Table S4 - Excel Table with all substrates including additional categorical information.
- Suppl Table S5 - Output tables of the Fisher Exact Testing for enrichment within the substrate populations for experiments M1, M2, M3.
