# Supplementary material for: A SILAC-based Approach Identifies Substrates of Caspase-dependent Cleavage upon TRAIL-induced Apoptosis
Source: Mol Cell Proteomics. 2013 Jan 13;12(5):1436–50. doi: 10.1074/mcp.M112.024679 (PMC3650350; doi:10.1074/mcp.M112.024679)
Supplement: Suppl Table S1 [file supp_M112.024679_mcp.M112.024679-6.pdf]

Table of Content:

1. Supplemental Figures

Figure S1
Background information on the experiments and algorithm.
Figure S2
Characterization of the substrate population.
Figure S3
Investigation of the uncleaved ratio distribution of cleaved substrates.
Figure S4
STRING analysis of known and novel substrates.
Figure S5
3D cleavage plots for components of the condensin I complex.
Figure S6
Cleavage of proteasomal proteins.

2. Supplemental Information

Instructions on how to use MaxQB for visualizing and extracting cleavage information.

3. Supplemental Tables

Supplemental Table S1:
Excel Table containing all substrates including 3D cleavage plots, cleavage statistics, uncleaved and cleaved ratios, sequence coverages and explicit site information.

Supplemental Table S2:
List of all known human cleavage substrates as defined within the text.

Supplemental Table S3:
Table containing substrates with cleavage site information (related to Figure 5C).

Supplemental Table S4:
Excel Table with all substrates including additional categorical information.

Supplemental Table S5:
Output tables of the Fisher Exact Testing for enrichment within the substrate populations for experiments M1, M2, M3.

(A)

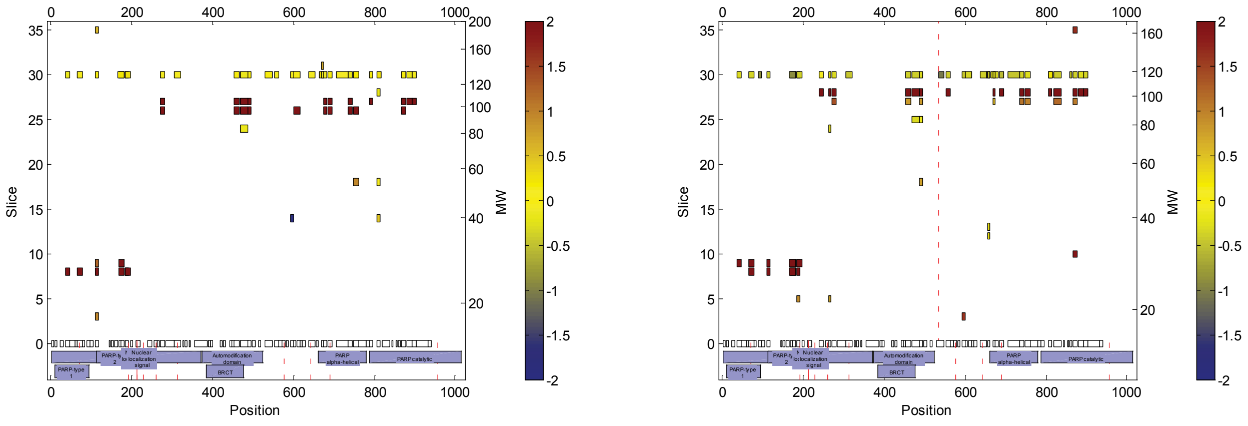

(B)

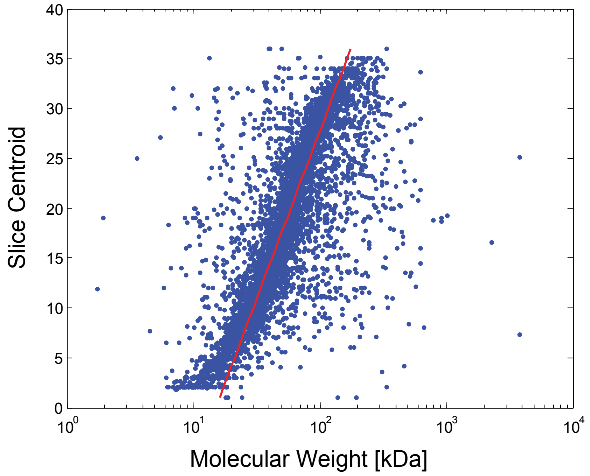

Figure S1: Background information on the experiments and algorithm.
A. Forward and reverse SILAC labeling. Both forward and reverse labeling resulted in the same ratio pattern for the cleaved substrate PARP1. **B.** Correlation of slice numbers to molecular weights. Based on all detected proteins of an experiment we calibrated approximate molecular weight regions along the gel. The red line is the regression line for the calculation of the right y-axis in the 3D cleavage plot.

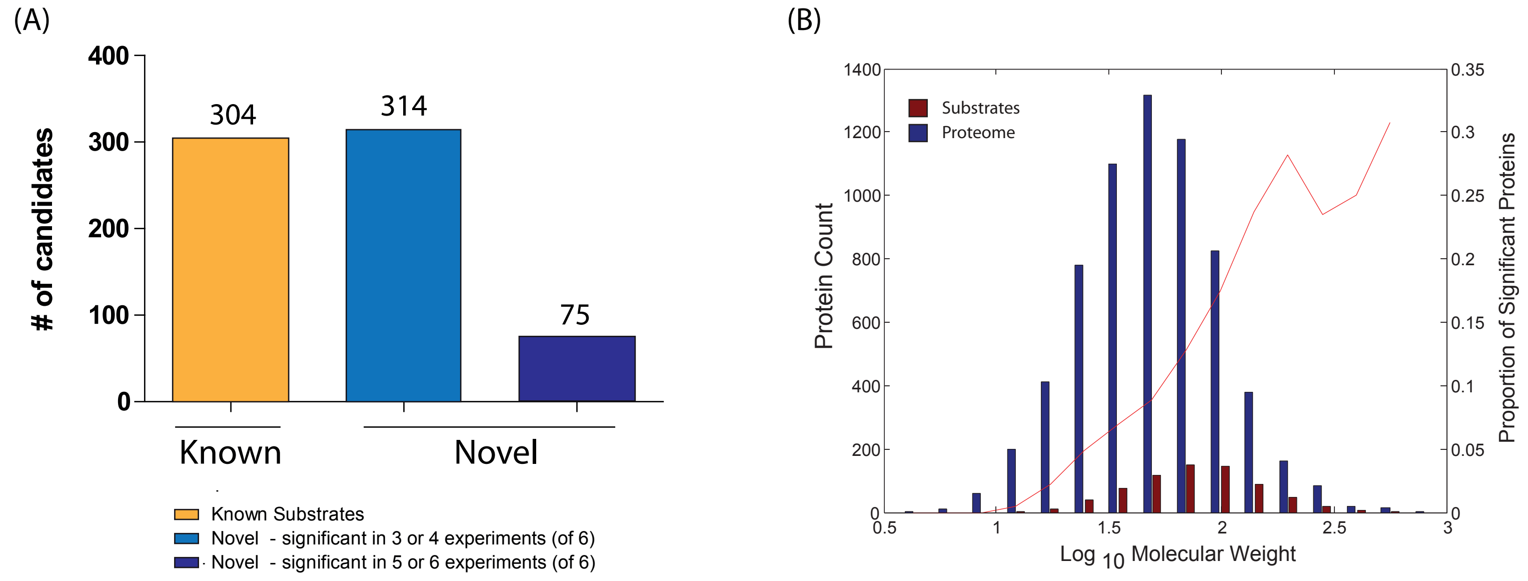

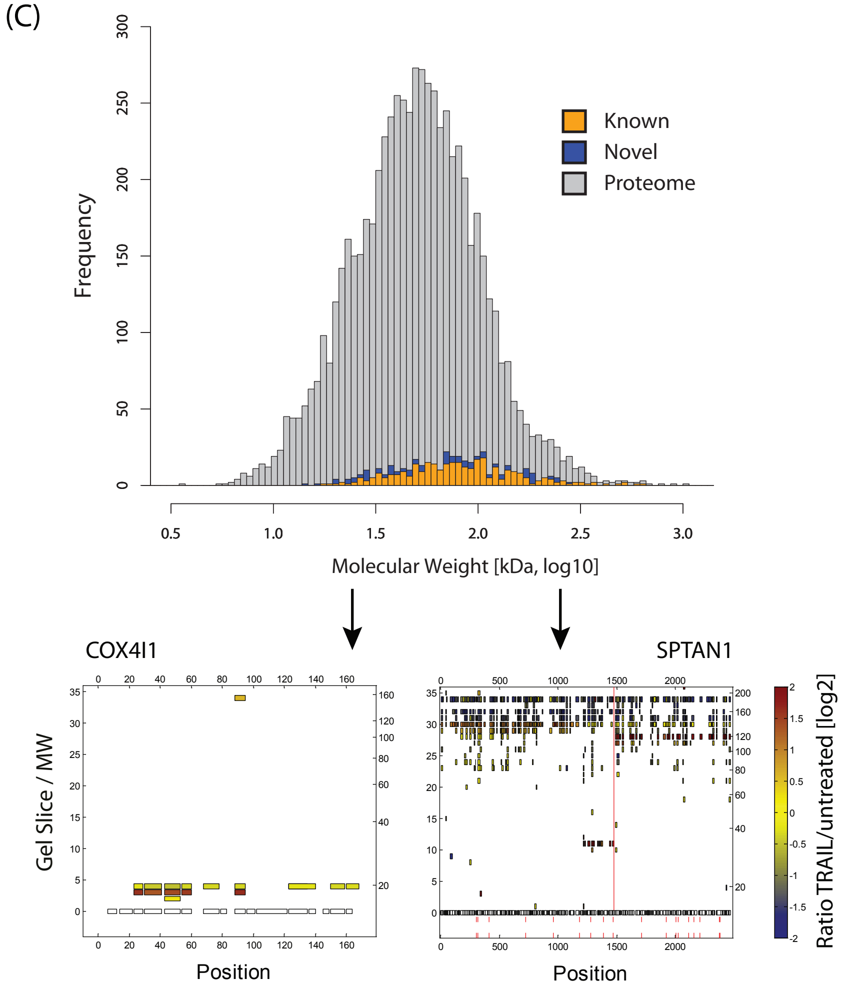

Figure S2: Characterization of the substrate population.

A. Characterization of identified substrates. Numbers of known and novel substrates are depicted. Novel substrates are subdivided depending on their number of significant identifications as cleavage substrate in all six experiments. Only substrates identified significantly at least three times are considered for the analysis. **B.** Coverage of the molecular weight region by the cleaved substrates. Whole proteome data as well as substrates are plotted. The red line indicates the proportion of significant substrates within the different molecular weight regions in comparison to the whole proteome. **C.** Distribution of molecular weights spanned by the whole proteome experiment and by the cleavage substrates. Proteome data are plotted according to their molecular weight, underlying known and novel substrates are highlighted. Substrates are generally detected between 20 kDa and 250 kDa. Representative examples of the lower and higher molecular weight regions are depicted.

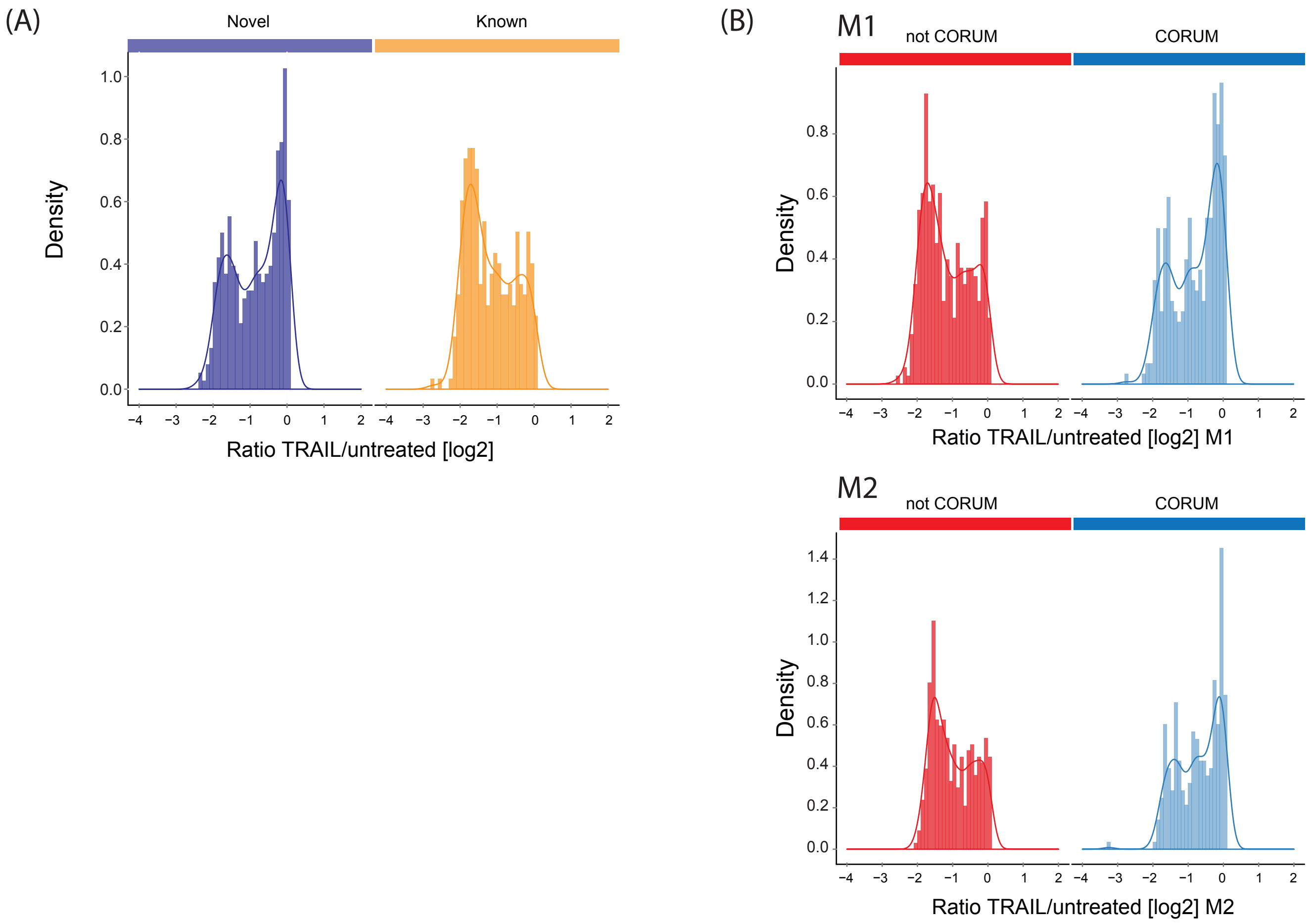

Figure S3: Investigation of the uncleaved ratio distribution of cleaved substrates.
A. Uncleaved ratio distribution of all cleaved substrates split for novel and known substrates. The uncleaved ratio distribution indicates a tendency of novel substrates to be located in the right hand peak in comparison to the population of known substrates. **B.** Separation of the uncleaved ratio population for CORUM and non-CORUM substrates within the experiments M1 and M2. Substrates annotated to CORUM show tendencies to be located in the right hand peak.

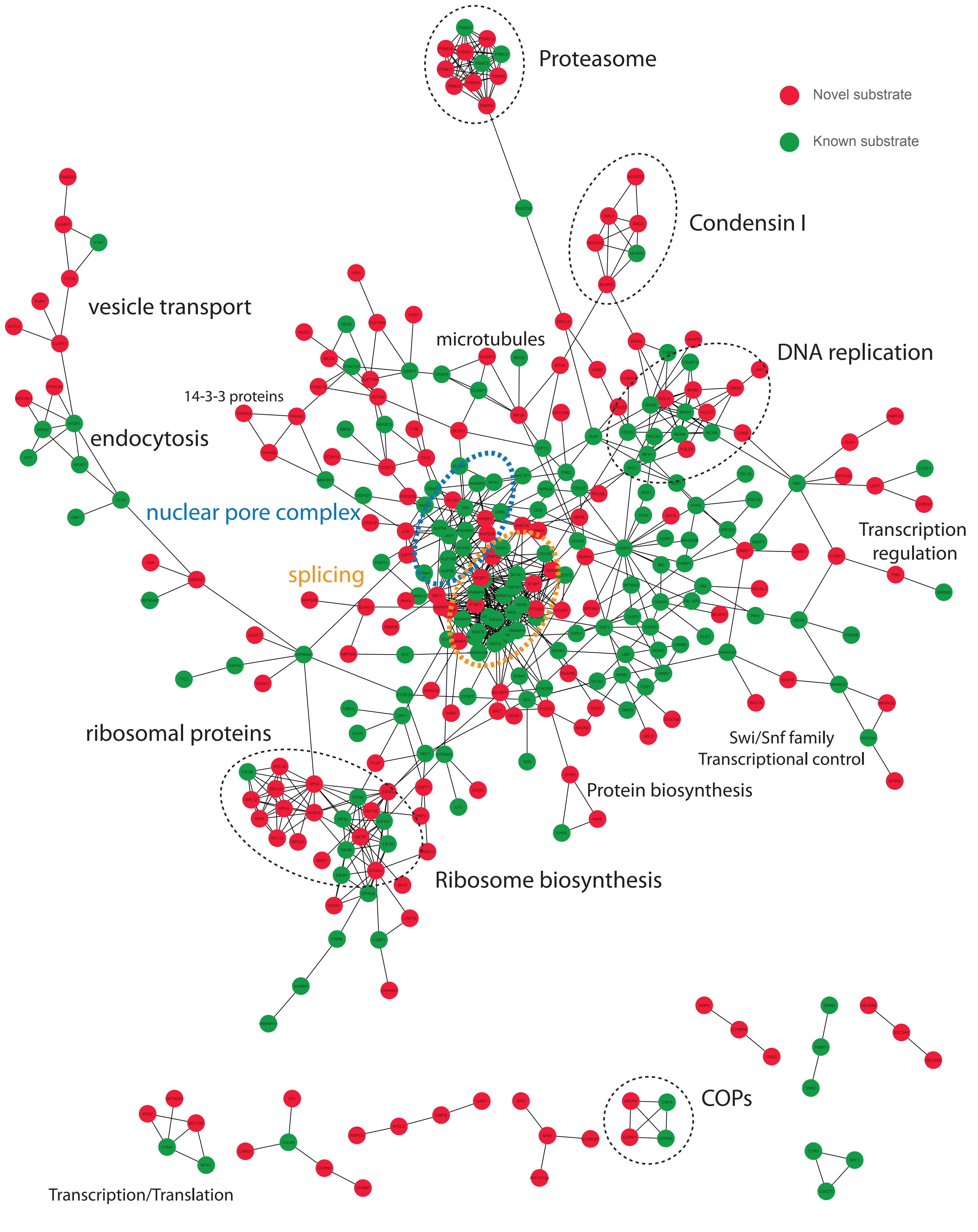

Supplemental Figure S4, Stoehr et al.

Figure S4: STRING analysis of known and novel substrates.
Substrates were uploaded to STRING and interactions were represented in the Cytoscape environment. Novel substrates are indicated in red, known substrates in green. Categories spanned by several interacting proteins are named within the graphic and encircled for clarity if necessary.

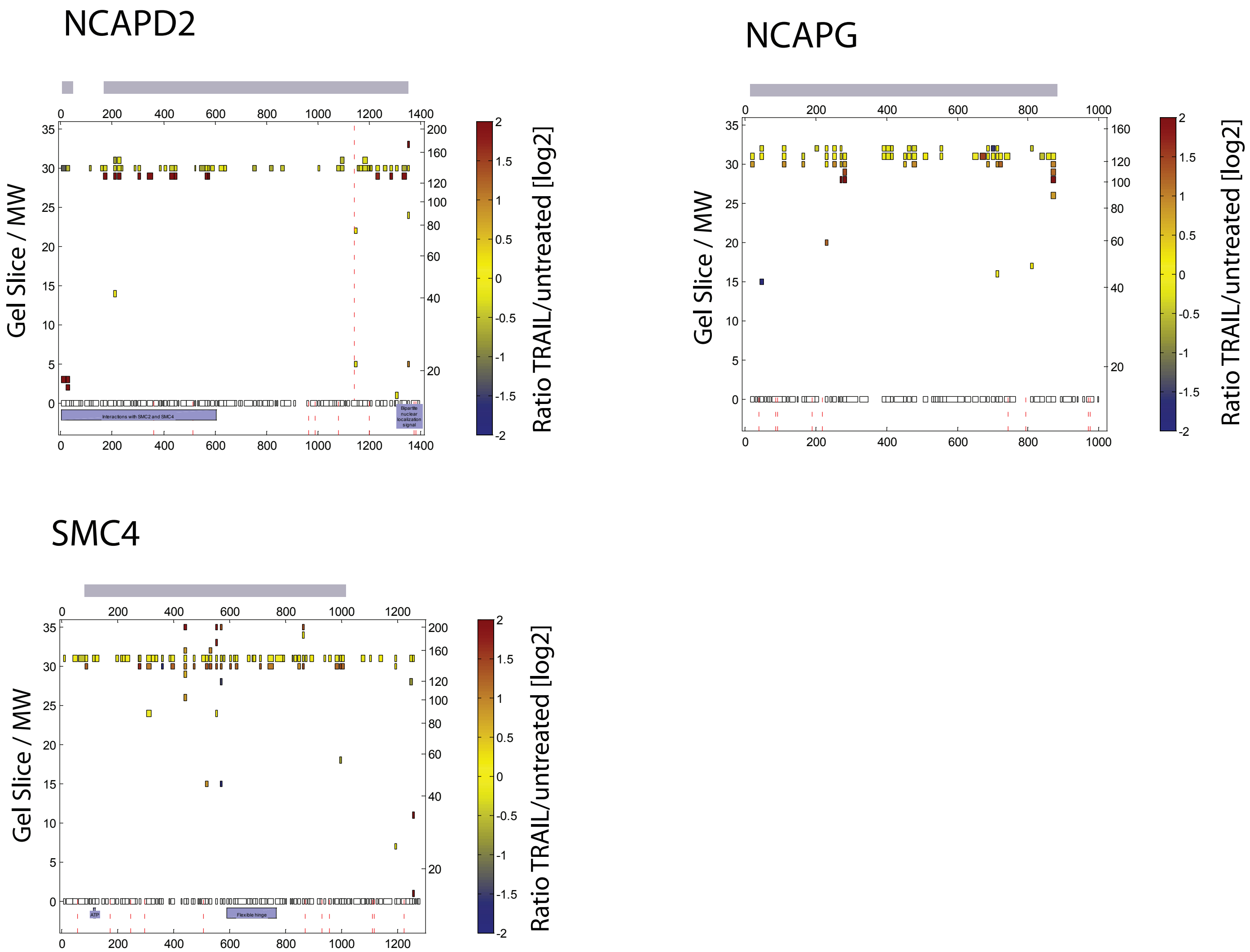

Figure S5: 3D cleavage plots for components of the condensin I complex.
Components from the condensin I complex not displayed in Fig. 7A are plotted. All proteins are cleavage substrates, cleavage fragments are indicated on top of the graph with grey bars.

19S regulatory particle (RP)

PSMD12,RPN5

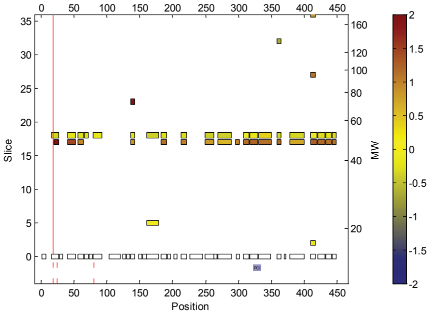

PSMD3,RPN3

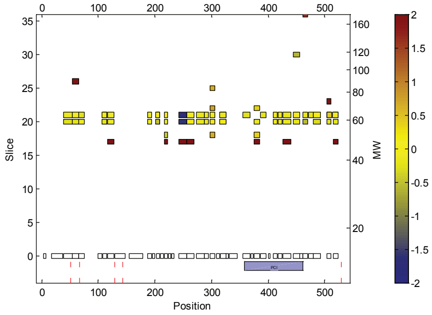

PSMD4,RPN10

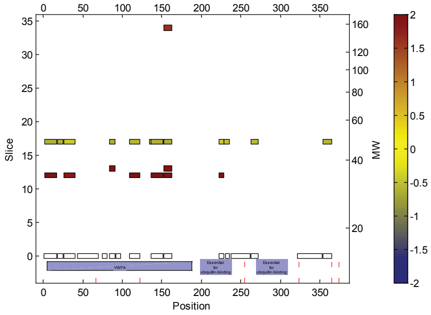

PSMD1,RPN2

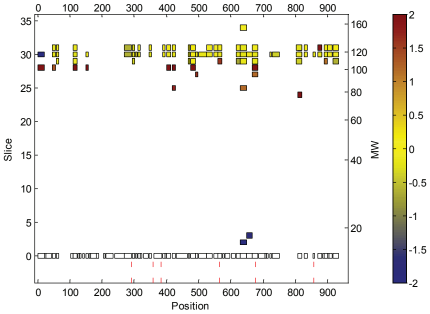

PSMC2,RPT1

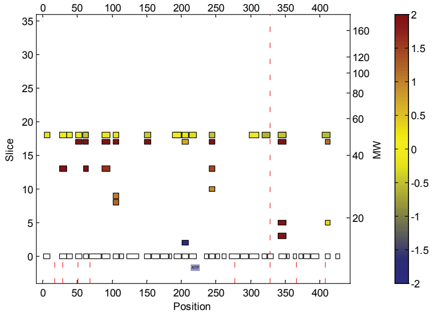

PSMC3,RPT5

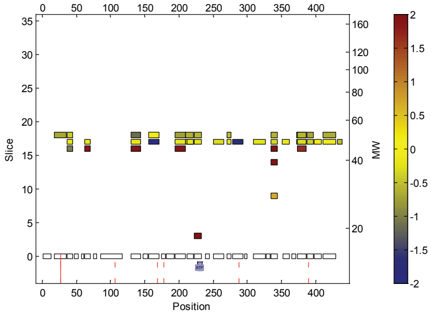

PSMC1,RPT2

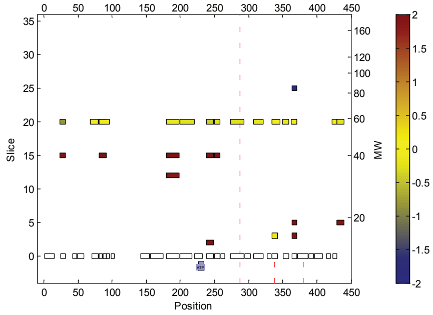

PSMC5,RPT6

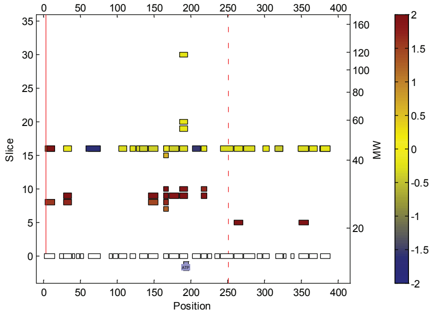

PSMC6,RPT4

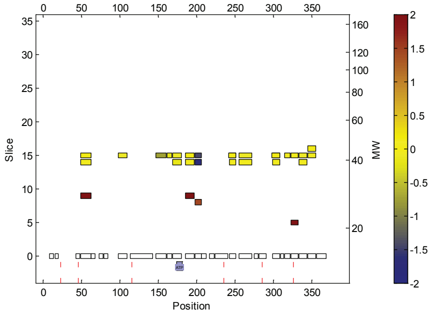

PA-28-gamma

PSME3

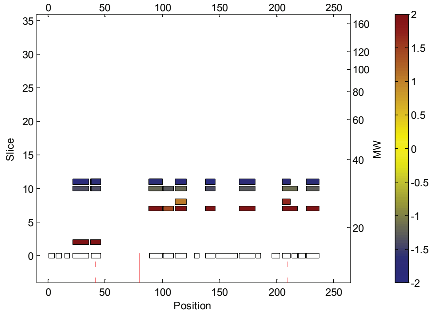

Core particle (20S proteasome)

PSMA5

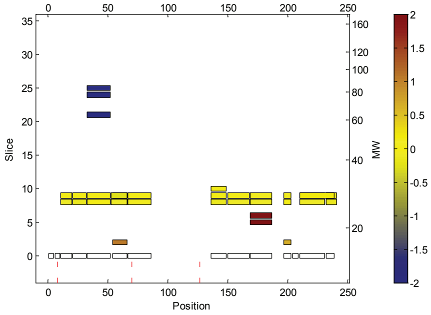

Supplemental Figure S6, Stoehr et al.

Figure S6: Cleavage of proteasomal proteins.
Cleaved proteins from the proteasome complex are depicted via their 3D cleavage plots. Proteins from both the 19S regulatory particle as well as the 20S core particle were identified as cleaved substrates. Most substrates show low stoichiometries in their uncleaved region.

Supplemental Information

Instructions on how to use MaxQB for visualizing and extracting cleavage information.

Starting from the project page, each of the experiments can be selected (1). Moreover, a list of proteins with an explicit cleavage information is provided (2).

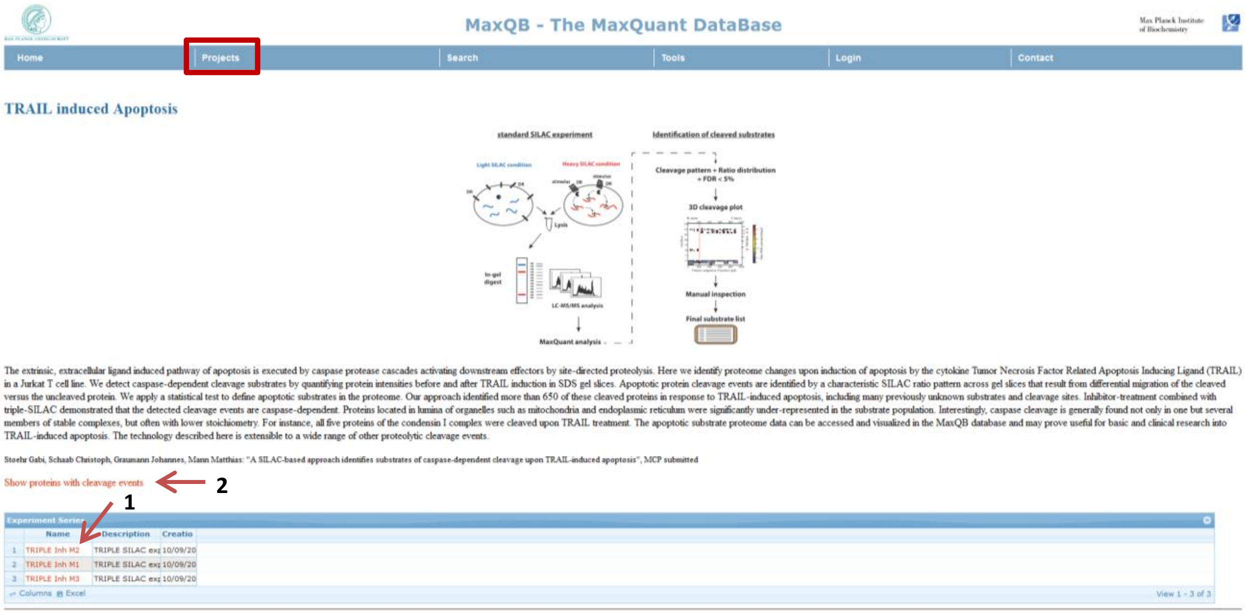

For each experiment general information on the experiment is provided.

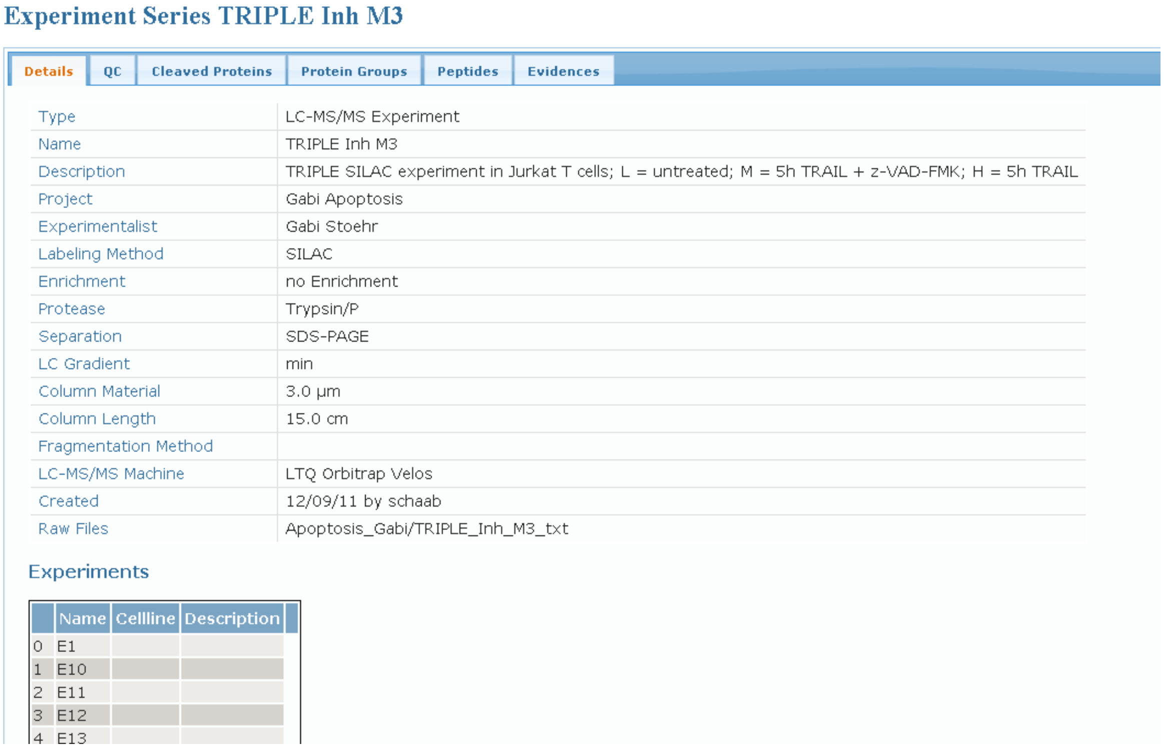

A list of the cleaved proteins can be accessed via the “Cleaved Proteins” tab including cleavage information on the single entries (p-value, FDR, ratio of cleaved peptides, …) (1).

Specific proteins can be searched by first clicking on the “Search” button at the bottom of the table (2). Each column can then be search for a specific term.

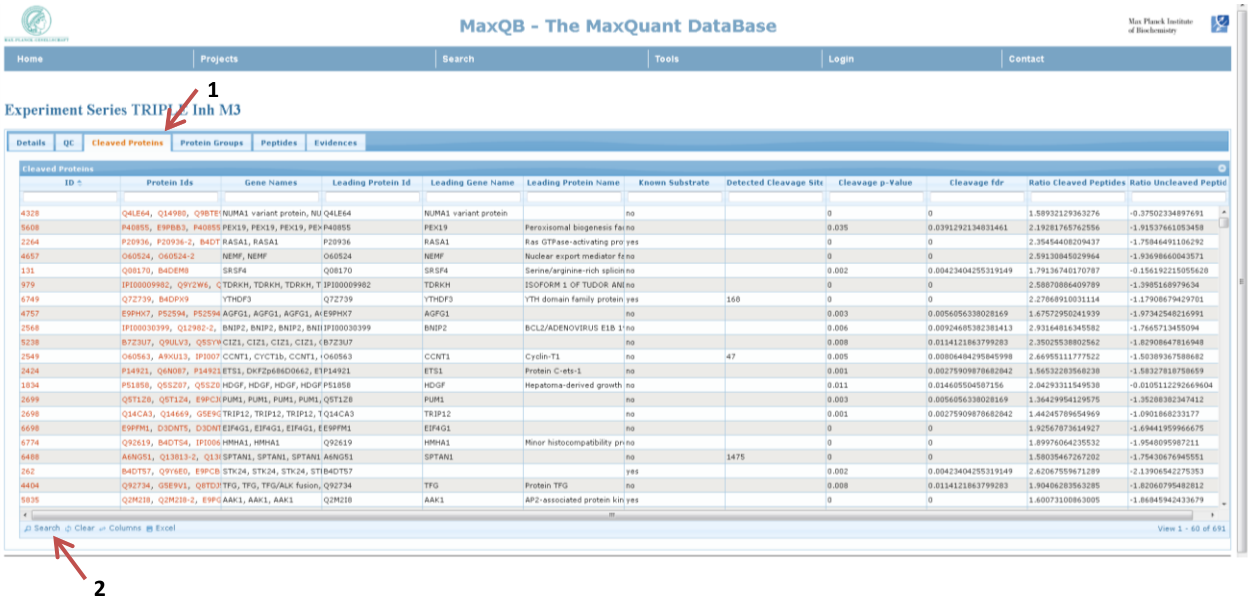

When selecting one ID in the “Cleaved proteins” table, further information about the protein within the experiment is provided.

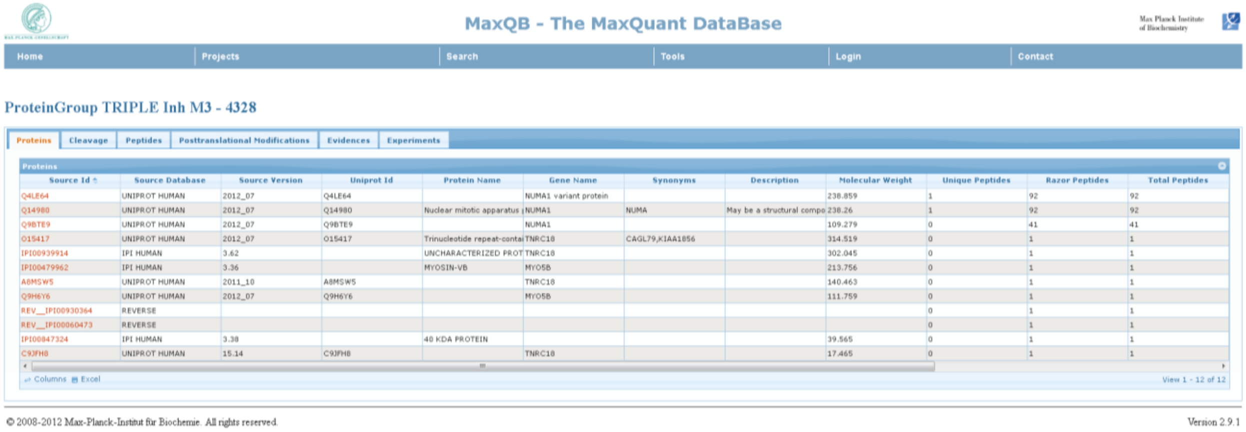

The “Cleavage” table contains all information about the cleavage of this protein within the specific experiment including statistics of the cleavage and the 3D cleavage plot.

ProteinGroup TRIPLE Inh M3 - 5633

Proteins | **Cleavage** | Peptides | Posttranslational Modifications | Evidences | Experiments

Source Id	P09874
Gene Name	PARP1
Protein Name	Poly [ADP-ribose] polymerase 1
Known Substrate	yes
Detected Cleavage Sites	
Cleavage p-Value	0,0000
Cleavage fdr	0,0000
Ratio Cleaved Peptides	1,68
Ratio Uncleaved Peptides	-1,93

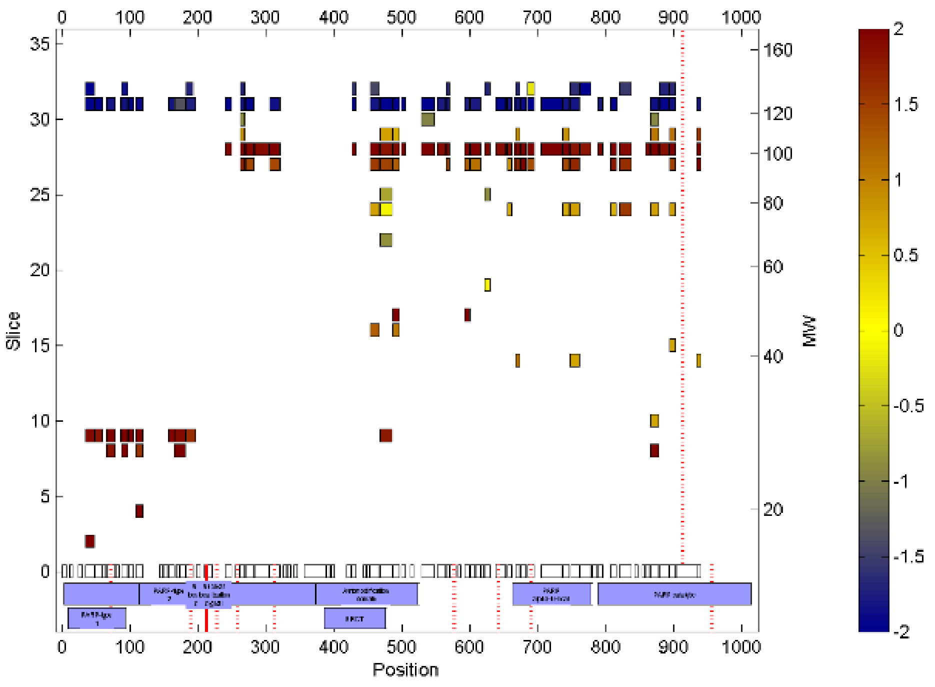

Selecting the Source Id in the “Proteins” tab or by selecting the Protein Id at the very beginning leads to the general protein information.

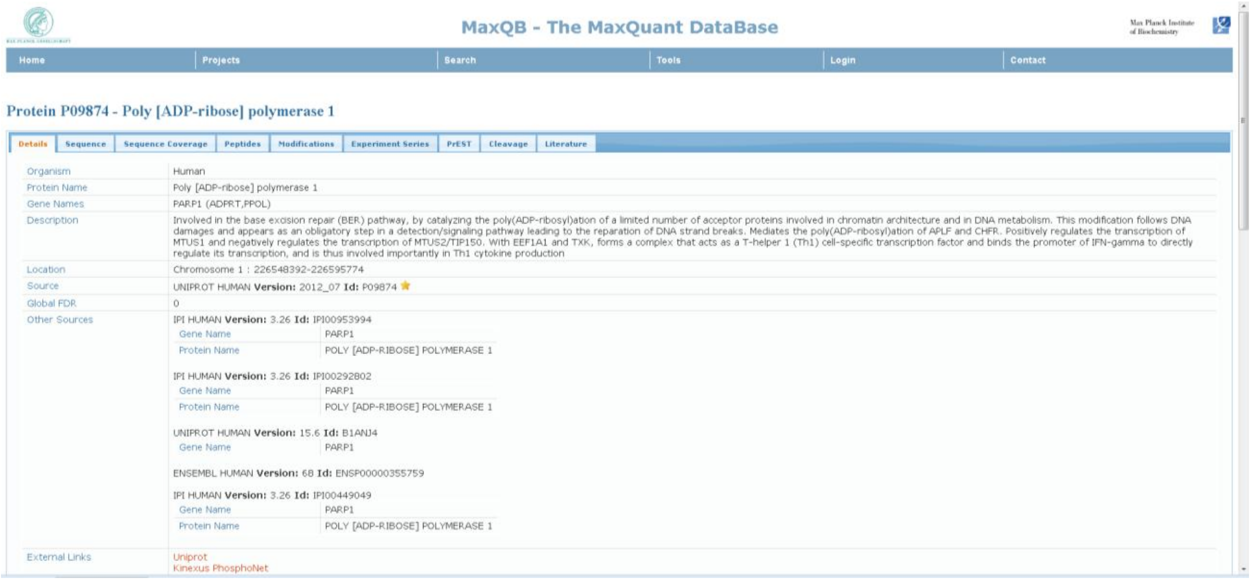

Information as e.g. general sequence coverages over all stored data can be accessed (for more information on general MaxQB features refer to the publication on MaxQB: Schaab et al., MCP, 2012).

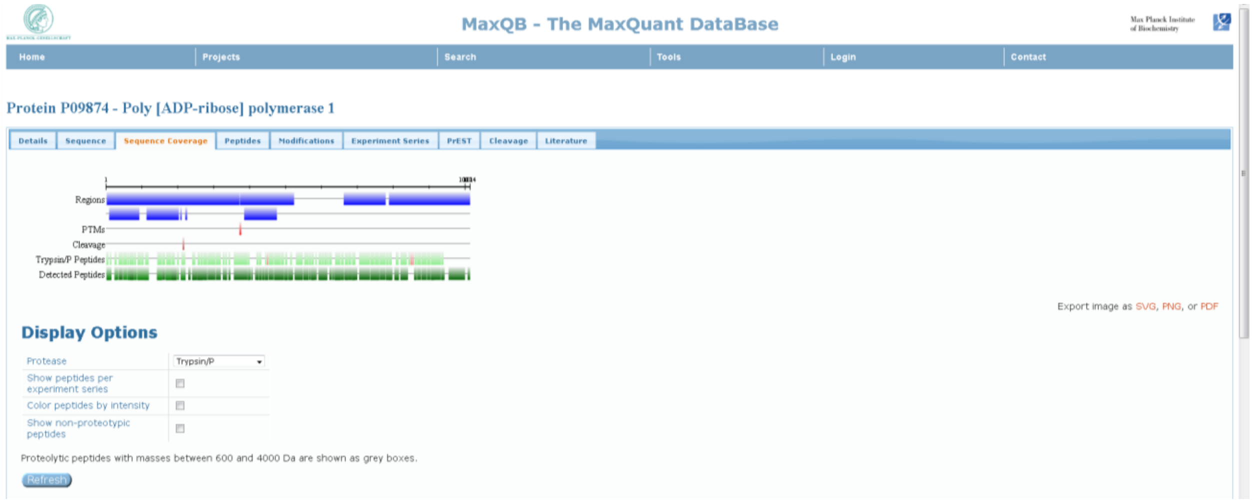

Again cleavage information can be accessed, this time, not based on a single experiment, but on all experiments. On top, information from literature about known cleavage sites is displayed. The hyperlink guides to the reference. Below, information derived from our experiments is displayed for all experiments – the 3D cleavage plot, cleavage site information as well as statistics on the cleavage per experiment.

Protein P09874 - Poly [ADP-ribose] polymerase 1

Details | Sequence | Sequence Coverage | Peptides | Modifications | Experiment Series | PrEST | Cleavage | Literature

Cleavage Sites from Literature

Pos	Source
214	CASBAH

Cleavage Sites determined by Stoehr et al.

Experiment Series	TRIPLE Inh M1
Protein Group	5518
Detected Cleavage Sites	
Cleavage p-Value	0,0000
Cleavage fdr	0,0000
Ratio Cleaved Peptides	1,65
Ratio Uncleaved Peptides	-1,95

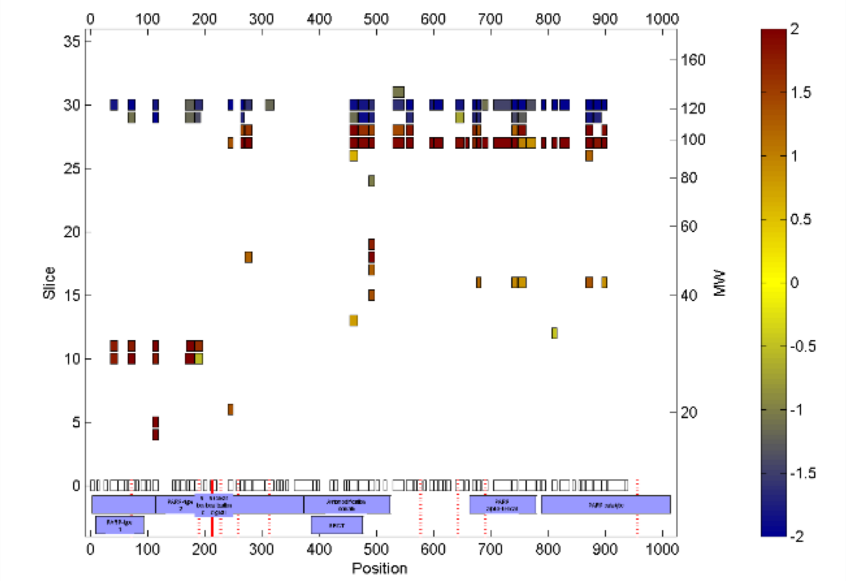

Proteins can also be searched directly via the “Search -> Proteins” function. By selecting the Source Id, general protein information will be obtained. In case the protein is known as cleavage substrate but was not identified by our approach, the information about the reference will still be provided in the “Cleavage” tab. In case no “Cleavage” tab is provided, the protein is not known to be a cleavage substrate.

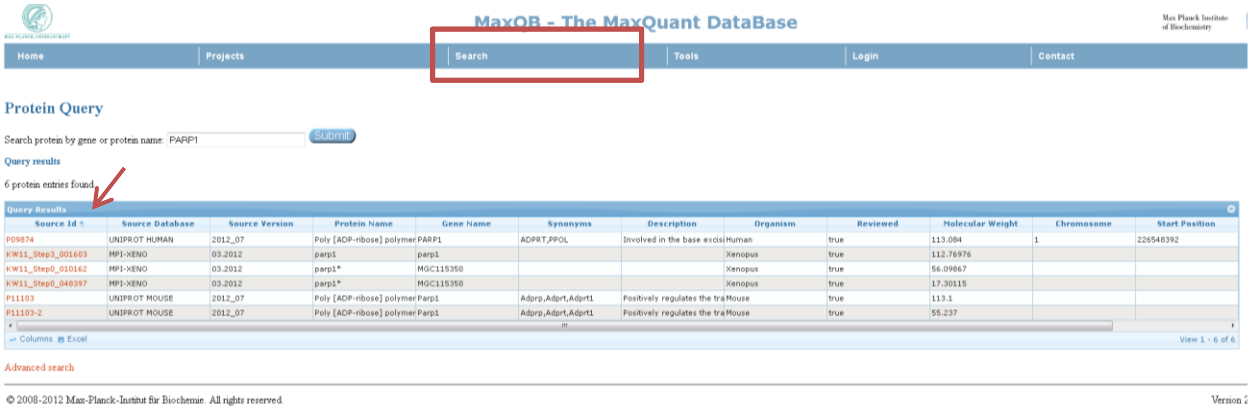

Source Id	Source Database	Source Version	Protein Name	Gene Name	Synonyms	Description	Organism	Reviewed	Molecular Weight	Chromosome	Start Position
P09874	UNIPROT HUMAN	2012_07	Poly [ADP-ribose] polymer	PARP1	ADPRT,PPOL	Involved in the base excisi	Human	true	113.084	1	226548392
KW11_Step3_001603	MPI-XENO	03.2012	parp1	parp1			Xenopus	true	112.76976		
KW11_Step0_010162	MPI-XENO	03.2012	parp1*	MGC115350			Xenopus	true	56.09867		
KW11_Step0_048397	MPI-XENO	03.2012	parp1*	MGC115350			Xenopus	true	17.30115		
P11103	UNIPROT MOUSE	2012_07	Poly [ADP-ribose] polymer	Parp1	Adprp,Adprt,Adprt1	Positively regulates the tra	Mouse	true	113.1		
P11103-2	UNIPROT MOUSE	2012_07	Poly [ADP-ribose] polymer	Parp1	Adprp,Adprt,Adprt1	Positively regulates the tra	Mouse	true	55.237		
